# Selection and validation of reference genes for quantitative Real-Time PCR in *Arabis alpina*

**DOI:** 10.1371/journal.pone.0211172

**Published:** 2019-03-04

**Authors:** Lisa Stephan, Vicky Tilmes, Martin Hülskamp

**Affiliations:** 1 Botanical Institute, Biocenter, Cologne University, Cologne, Germany; 2 Department of Plant Developmental Biology, Max Planck Institute for Plant Breeding Research, Cologne, Germany; Instituto de Biologia Molecular y Celular de Plantas, SPAIN

## Abstract

*Arabis alpina* is a perennial arctic-alpine plant and an upcoming model organism for genetics and molecular biology for the Brassicaceae family. One essential method for most molecular approaches is the analysis of gene expression by reverse-transcription quantitative Real-Time PCR (RT-qPCR). For the normalisation of expression data in RT-qPCR experiments, it is essential to use reliable reference genes that are not affected under a wide range of conditions. In this study we establish a set of 15 *A*. *alpina* reference genes that were tested under different conditions including cold, drought, heat, salt and gibberellic acid treatments. Data analyses with geNORM, BestKeeper and NormFinder revealed the most stable reference genes for the tested conditions: *RAN3*, *HCF* and *PSB33* are most suitable for cold treatments; *UBQ10* and *TUA5* for drought; *RAN3*, *PSB33* and *EIF4a* for heat; *CAC*, *TUA5*, *ACTIN 2* and *PSB33* for salt and *PSB33* and *TUA5* for gibberellic acid treatments. *CAC* and *ACTIN 2* showed the least variation over all tested samples. In addition, we show that two reference genes are sufficient to normalize RT-qPCR data under our treatment conditions. In future studies, these reference genes can be used for an adequate normalisation and thus help to generate high quality RT-qPCR data in *A*. *alpina*.

## Introduction

Recently, *A*. *alpina* has been established as a new model system in the Brassicaceae family [1,2]. It is native to mountains and arctic-alpine habitats [3,4] and combines several features enabling genetic and molecular studies: it is diploid, self-fertile, has a small and sequenced genome and can be transformed with *Agrobacterium tumefaciens* [1]. *A*. *alpina* has an evolutionary distance to *A*. *thaliana* of about 26 to 40 million years [4,5]. This facilitates functional comparisons of biological processes, as orthologous genes can be identified by sequence similarity and synteny [6].

Most molecular studies require quantitative analyses of the expression of genes of interest by reverse-transcription quantitative Real-Time PCR (RT-qPCR). For proper comparisons of expression levels, the expression data of the genes under study are normalized using genes as a reference that show no or very little variation under different conditions. In 2009, the Minimum Information for Publication of Quantitative Real-Time PCR Experiments (MIQE) guidelines were published, with the aim to provide a consensus on correct performance and interpretation of RT-qPCR experiments [7]. These guidelines should ensure that the normalisation enables the comparison of transcripts in different samples by correcting variations in yields of extraction and reverse transcription and the efficiency of amplification. A pre-requisite for any RT-qPCR analysis are suitable primer sets for reference genes that are thoroughly tested. These need to fulfil various requirements: primers should create a specific amplicon of 80 to 200 bp, without creating primer dimers. The amplification should be carried out with close to 100% efficiency and show a linear standard curve with a correlation of more than 0.99. In general, there should be minimal variation between replicates, indicating consistent performance of the primers.

In this study we established primer pairs for 15 reference genes that can be used for future RT-qPCR studies in *A*. *alpina*: *ADENOSINE TRIPHOSPHATASE* (*ATPase*), *THIOREDOXIN*, *HIGH CHLOROPHYLL FLUORESCENCE 164* (*HCF*), *EUKARYOTIC TRANSLATION INITIATION FACTOR 4A1* (*EIF4a*), *RAN GTPASE 3* (*RAN3*), *UBIQUITIN 10* (*UBQ10*), *ACTIN 2*, *PHOTOSYSTEM B PROTEIN 33* (*PSB33*), *HISTONE H3*, *NAD(P)H PLASTOQUINONE DEHYDROGENASE COMPLEX SUBUNIT O* (*NdhO*), *TUBULIN ALPHA 5* (*TUA5*), *18s RIBOSOMAL RNA* (*18srRNA*), *CLATHRIN ADAPTOR COMPLEX MEDIUM SUBUNIT* (*CAC*), *SAND family protein* (*SAND*) and *HEAT SHOCK PROTEIN 81*.*2/90* (*HSP81*.*2/90*). The primers were thoroughly tested, and reference genes were evaluated for variations in their expression under different conditions including cold, drought, heat and salt in whole seedlings and gibberellic acid (GA) treatments in leaves. Using genes specifically responding to the different treatment, we demonstrate the impact of normalization with our reference genes.

## Results

### Selection and validation of reference genes

To compile a set of suitable reference genes, we pursued three approaches: first, we selected *A*. *thaliana* genes that are known to show little variation under different conditions. Corresponding orthologs in *A*. *alpina* were then identified by sequence similarity and synteny. Second, we identified genes which show stable expression in *A*. *alpina* over an extended period of time. Third, we included a well-established reference gene from *A*. *alpina* from former studies. Thus, we created a set of 15 reference genes (Table 1), including nine orthologs to *A*. *thaliana* reference genes, five novel reference genes and *RAN3*, a known reference gene for RT-qPCR in *A*. *alpina* [1]. We first amplified the gene fragments and verified the amplicon by sequencing. Additionally, we analysed the melting curves to exclude unspecific products and/or primer dimers (S1 Fig).

**Table 1:** Candidate reference genes, primers and amplicons.

Name	Gene ID	Arabidopsis homolog	Primer sequence (5’ to 3’)		Amplicon length [bp]
*ATPase*	Aa_G9730	AT2G25610	F R	GCCAACCTTGTATGCGGGTTA GTTGGCCATGTTGCTTGTGC	176 **
*THIOREDOXIN*	Aa_G337160	AT5G03880	F R	GATTGGTCGTGCCGGAAAGG TCTCGAGCAATTCCTGTCGTTTG	188 **
*HCF*	Aa_G363210	AT4G37200	F R	AAGGTAATGTTGTCGGGAGGCT CGGCTCGGGCATGAGGAAT	96
*EIF4a*	Aa_G472320	AT3G13920	F R	CCAGCTTCTCCCACCCAAGA GCTCGTCACGCTTCACCAAG	122
*RAN3*	Aa_G442020	AT5G55190	F R	CACAGGAAAAACCACATTCGT CCATCCCTAAGACCACCAAAT	174 **
*UBQ10*	Aa_G41880	AT4G05320	F R	CGTCTCCGTGGTGGTTTCTA AAGGCCCCAAAACACAAACG	122
*ACTIN 2*	NA	AT3G18780	F R	AGCTGTTCTCTCCCTGTACG AACCCTCGTAGATTGGCACA	94 *
*PSB33*	Aa_G319470	AT1G71500	F R	TGGCGACCACTGCATCTTCA ATCGACGGTCACGACGGAGA	117
*HISTONE H3*	NA	AT5G65360	F R	CTCACGGAGAGCGACGGTTC GCAACTCGCGACGAAAGCAG	97
*NdhO*	Aa_G477070	AT1G74880	F R	GCGGCGAGGTCTTGGACATT TCGCTCGTAAACAAGTTTCTCAGACT	134
*TUA5*	NA	AT5G19780	F R	TGTGACCCGAGGCACGGAAA CCAGTAGGGCACCAGTCAACA	137**
*18srRNA*	NA	AT3G41768	F R	CTCCGATCCCGAAGGCCAAC CCTTAACTGGCCGGGTCGTG	141
*CAC*	Aa_G26240	AT5G46630	F R	TGGACAAGACCACCAATCCA CACTCGACCGTGTTGTAACC	113 *
*SAND*	Aa_G18160	AT2G28390	F R	TTGCAGGATTCGCATTGAGG TCCAAAGGGACCTCCTGTTC	178 **
*HSP81*.*2/90*	NA	AT5G56030	F R	CGTCTGGTGAGGCTCTTGGT AGCCTACGCTCCTCAAGGTACT	85

NA–not annotated (identified by nucleotide BLAST), F–forward primer, R–reverse primer, bp–base pair

* amplicon contains an intron on genomic level

**amplicon contains an intron on genomic level and at least one primer is spanning an exon-intron junction.

Subsequently, we determined the primer efficiencies and correlation coefficients to demonstrate the quality of the primer pairs (Table 2, S2 Fig). All reference gene primers displayed an efficiency between 96.42 and 107.01% and a correlation higher than 0.99.

**Table 2:** Primer efficiencies and correlation of reference gene candidates.

Gene	Efficiency [%]	R^2^
*ATPase*	98.33	-0.996
*THIOREDOXIN*	101.92	-0.992
*HCF*	99.98	-0.997
*EIF4a*	107.01	-0.992
*RAN3*	102.30	-0.998
*UBQ10*	102.52	-0.999
*ACTIN 2*	102.85	-0.999
*PSB33*	99.51	-1.000
*HISTONE H3*	99.14	-0.999
*NdhO*	94.34	-0.999
*TUA5*	98.32	-1.000
*18srRNA*	98.65	-1.000
*CAC*	98.07	-0.999
*SAND*	96.42	-0.999
*HSP81*.*2/90*	96.74	-0.999

Efficiency [%]–efficiency of the primers was calculated with $E[\%]=100\times {(10}^{\left(-\frac{1}{slope}\right)}-1)$, slope–average Cq values (y) and log10 values of six serial dilutions (x) were used to calculate the slope of a regression line with the formula $slope=\frac{\sum \left(x-\bar{x})(y-\bar{y}\right)}{{\sum \left(x-\bar{x}\right)}^{2}}$, R^2^ –correlation of the Cq values calculated with the formula $\rho_{x,y}=\frac{Cov(X,Y)}{\sigma_{x}\times \sigma_{y}}$.

### Expression stability of reference genes after treatments

**Fig 1:**
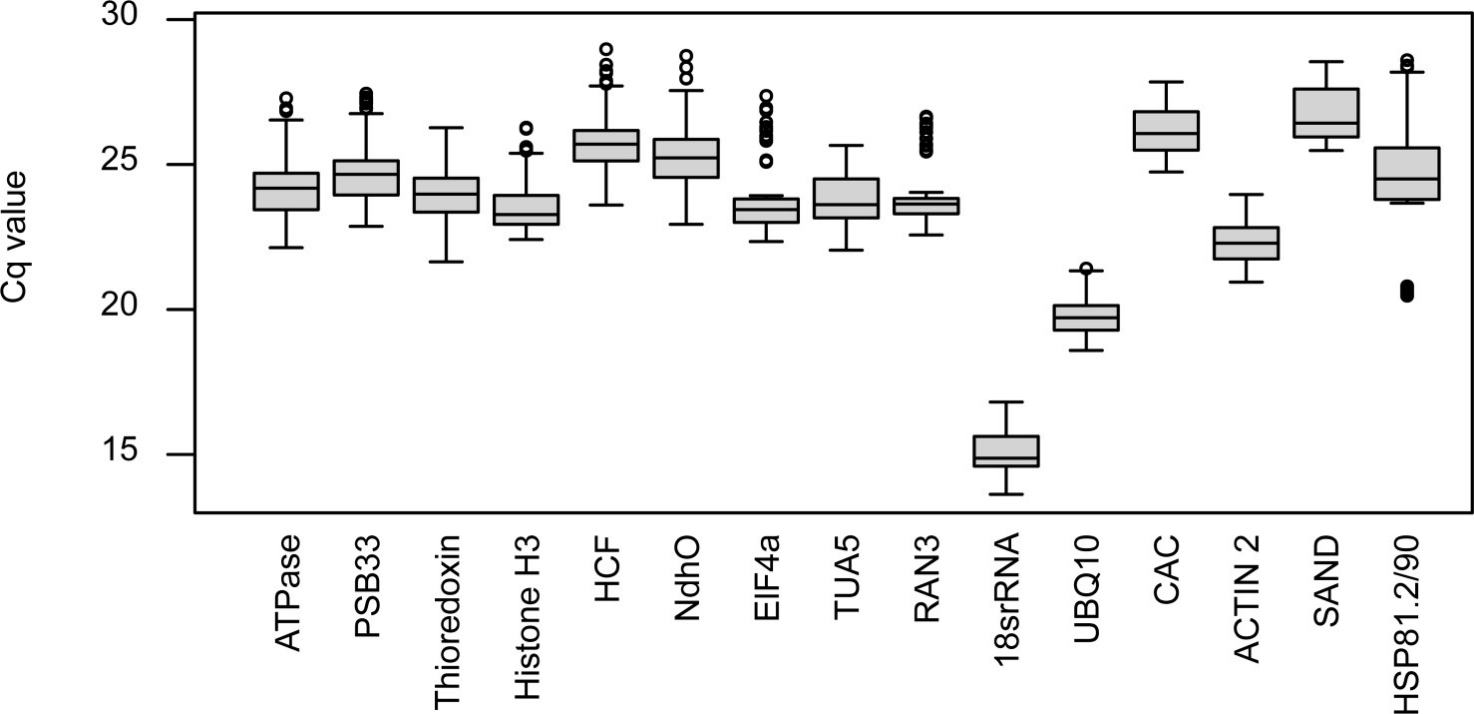
RT-qPCR Cq values of candidate reference genes in all treatments. The box includes all data points between the 25% quantile and the 75% quantile. The whiskers span all values below and above, excluding outliers which are displayed as dots. The median, which marks the 50% quantile, is displayed as a line.

The expression levels of the reference genes were detected as cycle quantification (Cq) values. The mean Cq values over all treatments ranged between 15.10 (*18srRNA*) and 26.68 (*SAND*). The *18srRNA*, *UBQ10* and *ACTIN 2* genes showed the highest expression/lowest Cq (Fig 1). Overall, Cq values for a single reference gene varied 4.30 on average, with a minimum variation of 2.82 (*UBQ10*) and a maximum variation of 8.12 (*HSP81*.*2/90*).

Next, we analysed the expression of the selected reference genes under different conditions, including cold, drought, heat, salt and gibberellic acid treatments (Table 3, S1 Table). The stability was calculated using three common statistical algorithms: NormFinder [8], BestKeeper [9] and geNorm [10]. These algorithms can be used to rank reference genes of a given set by their stability and determine the most stable genes for the tested conditions. In contrast to BestKeeper and NormFinder, geNorm additionally provides a cut off value (1.5), below which primer pairs are considered adequate for normalisation. All genes in all treatments, except for *HSP81*.*2/90* under heat stress conditions, met this criterion. Thus, all reference genes reported here can be considered suitable for a wide range of conditions. The most stable genes for cold treatments were *RAN3*, *HCF* and *PSB33*. According to the BestKeeper algorithm, *EIF4a* could be considered as a reference for cold treatments as well. However, geNorm advises against this gene, ranking it at position eight. After drought treatment, *UBQ10* and *TUA5* showed the least overall variation in the three methods. Here in particular, the results strongly differed between the algorithms. While *HISTONE H3* ranked first for BestKeeper, it was found to be least stable in the other two methods. On the contrary, *EIF4a* and *THIOREDOXIN* showed high stability in NormFinder and geNorm, but low values in BestKeeper. Under heat conditions, *RAN3*, *PSB33* and *EIF4a* were most stable over all algorithms and under salt conditions *CAC*, *TUA5*, *ACTIN 2* and *PSB33* performed best. Finally, under GA treatment, *PSB33* and *TUA5* had the least expression variation, while *THIOREDOXIN* showed stable expression only for NormFinder and geNorm. When taking all stress treatments into account, *THIOREDOXIN* and *CAC* showed the least variation. When considering all treatments, *CAC* and *ACTIN 2* showed the most stable expression (S2 Table).

**Table 3:** Ranking of gene expression stability under stress conditions and hormone stimuli. Genes were ranked using the three commonly used statistical algorithms NormFinder, BestKeeper and geNorm. The stability value describes the variance (NormFinder and geNorm) or standard deviation (BestKeeper).

	Rank	NormFinder		BestKeeper		geNorm	
		Gene	Stability	Gene	Stability	Gene	Stability
**Cold**	1	*HCF*	0.04	*RAN3*	1.11	*PSB33*	0.28
	2	*RAN3*	0.06	*EIF4a*	1.13	*RAN3*	0.28
	3	*PSB33*	0.07	*HCF*	1.14	*HCF*	0.29
	4	*EIF4a*	0.10	*NdhO*	1.16	*TUA5*	0.30
	5	*ATPase*	0.11	*UBQ10*	1.17	*ATPase*	0.31
	6	*TUA5*	0.12	*TUA5*	1.18	*THIOREDOXIN*	0.31
	7	*THIOREDOXIN*	0.14	*HISTONE H3*	1.18	*CAC*	0.31
	8	*NdhO*	0.15	*PSB33*	1.18	*EIF4a*	0.32
	9	*HISTONE H3*	0.15	*SAND*	1.19	*SAND*	0.33
	10	*CAC*	0.15	*THIOREDOXIN*	1.21	*NdhO*	0.35
	11	*SAND*	0.15	*ATPase*	1.21	*HISTONE H3*	0.36
	12	*UBQ10*	0.17	*CAC*	1.23	*ACTIN 2*	0.37
	13	*ACTIN 2*	0.22	*ACTIN 2*	1.26	*UBQ10*	0.38
	14	*18srRNA*	0.28	*18srRNA*	1.34	*18srRNA*	0.45
	15	*HSP81*.*2/90*	0.63	*HSP81*.*2/90*	1.75	*HSP81*.*2/90*	0.92
**Drought**	1	*UBQ10*	0.05	*HISTONE H3*	1.11	*UBQ10*	0.19
	2	*EIF4a*	0.07	*TUA5*	1.14	*EIF4a*	0.20
	3	*THIOREDOXIN*	0.08	*NdhO*	1.18	*THIOREDOXIN*	0.21
	4	*CAC*	0.08	*HCF*	1.20	*CAC*	0.21
	5	*TUA5*	0.08	*UBQ10*	1.20	*TUA5*	0.22
	6	*SAND*	0.09	*SAND*	1.22	*ATPase*	0.22
	7	*ATPase*	0.10	*CAC*	1.22	*HCF*	0.22
	8	*HCF*	0.10	*HSP81*.*2/90*	1.22	*SAND*	0.22
	9	*NdhO*	0.10	*THIOREDOXIN*	1.23	*NdhO*	0.23
	10	*ACTIN 2*	0.12	*ATPase*	1.24	*ACTIN 2*	0.24
	11	*RAN3*	0.12	*18srRNA*	1.24	*RAN3*	0.24
	12	*PSB33*	0.15	*EIF4a*	1.25	*PSB33*	0.26
	13	*18srRNA*	0.17	*ACTIN 2*	1.27	*18srRNA*	0.30
	14	*HSP81*.*2/90*	0.19	*RAN3*	1.30	*HSP81*.*2/90*	0.32
	15	*HISTONE H3*	0.25	*PSB33*	1.30	*HISTONE H3*	0.39
**Heat**	1	*EIF4a*	0.04	*RAN3*	1.09	*EIF4a*	0.47
	2	*RAN3*	0.04	*EIF4a*	1.12	*ATPase*	0.48
	3	*PSB33*	0.04	*PSB33*	1.14	*RAN3*	0.48
	4	*HCF*	0.04	*HCF*	1.15	*THIOREDOXIN*	0.48
	5	*THIOREDOXIN*	0.08	*THIOREDOXIN*	1.18	*HISTONE H3*	0.50
	6	*ATPase*	0.09	*ATPase*	1.20	*PSB33*	0.51
	7	*NdhO*	0.12	*NdhO*	1.20	*HCF*	0.52
	8	*HISTONE H3*	0.18	*HISTONE H3*	1.22	*ACTIN 2*	0.52
	9	*18srRNA*	0.22	*ACTIN 2*	1.28	*NdhO*	0.55
	10	*ACTIN 2*	0.24	*18srRNA*	1.28	*CAC*	0.58
	11	*CAC*	0.32	*CAC*	1.37	*TUA5*	0.60
	12	*TUA5*	0.35	*TUA5*	1.39	*18srRNA*	0.61
	13	*UBQ10*	0.40	*UBQ10*	1.47	*SAND*	0.76
	14	*SAND*	0.49	*SAND*	1.61	*UBQ10*	0.87
	15	*HSP81*.*2/90*	1.74	*HSP81*.*2/90*	5.22	*HSP81*.*2/90*	2.52
**Salt**	1	*CAC*	0.11	*TUA5*	1.16	*CAC*	0.55
	2	*THIOREDOXIN*	0.12	*CAC*	1.18	*PSB33*	0.57
	3	*ACTIN 2*	0.15	*18srRNA*	1.19	*ACTIN 2*	0.57
	4	*PSB33*	0.15	*ACTIN 2*	1.19	*TUA5*	0.57
	5	*TUA5*	0.17	*PSB33*	1.22	*THIOREDOXIN*	0.57
	6	*NdhO*	0.19	*SAND*	1.24	*HISTONE H3*	0.62
	7	*HISTONE H3*	0.22	*THIOREDOXIN*	1.24	*NdhO*	0.63
	8	*SAND*	0.27	*NdhO*	1.25	*UBQ10*	0.64
	9	*UBQ10*	0.29	*HISTONE H3*	1.25	*SAND*	0.66
	10	*HCF*	0.37	*UBQ10*	1.34	*18srRNA*	0.71
	11	*18srRNA*	0.37	*HCF*	1.4	*HCF*	0.73
	12	*ATPase*	0.41	*ATPase*	1.59	*ATPase*	0.83
	13	*HSP81*.*2/90*	0.69	*HSP81*.*2/90*	1.93	*HSP81*.*2/90*	1.08
	14	*EIF4a*	0.75	*EIF4a*	1.95	*EIF4a*	1.18
	15	*RAN3*	0.93	*RAN3*	2.43	*RAN3*	1.4
**GA**	1	*TUA5*	0.05	*SAND*	1.15	*TUA5*	0.21
	2	*THIOREDOXIN*	0.06	*RAN3*	1.15	*PSB33*	0.22
	3	*CAC*	0.07	*PSB33*	1.15	*THIOREDOXIN*	0.22
	4	*PSB33*	0.08	*TUA5*	1.16	*CAC*	0.22
	5	*NdhO*	0.09	*EIF4a*	1.17	*ACTIN 2*	0.23
	6	*ACTIN 2*	0.09	*CAC*	1.17	*NdhO*	0.23
	7	*HCF*	0.09	*ACTIN 2*	1.18	*HCF*	0.24
	8	*RAN3*	0.11	*HCF*	1.18	*RAN3*	0.24
	9	*EIF4a*	0.12	*THIOREDOXIN*	1.19	*EIF4a*	0.25
	10	*SAND*	0.13	*NdhO*	1.20	*SAND*	0.26
	11	*ATPase*	0.13	*ATPase*	1.22	*ATPase*	0.27
	12	*UBQ10*	0.15	*HISTONE H3*	1.28	*UBQ10*	0.29
	13	*HSP81*.*2/90*	0.16	*UBQ10*	1.32	*HSP81*.*2/90*	0.30
	14	*HISTONE H3*	0.20	*HSP81*.*2/90*	1.34	*HISTONE H3*	0.34
	15	*18srRNA*	0.35	*18srRNA*	1.37	*18srRNA*	0.53

### Optimal number of reference genes

**Fig 2:**
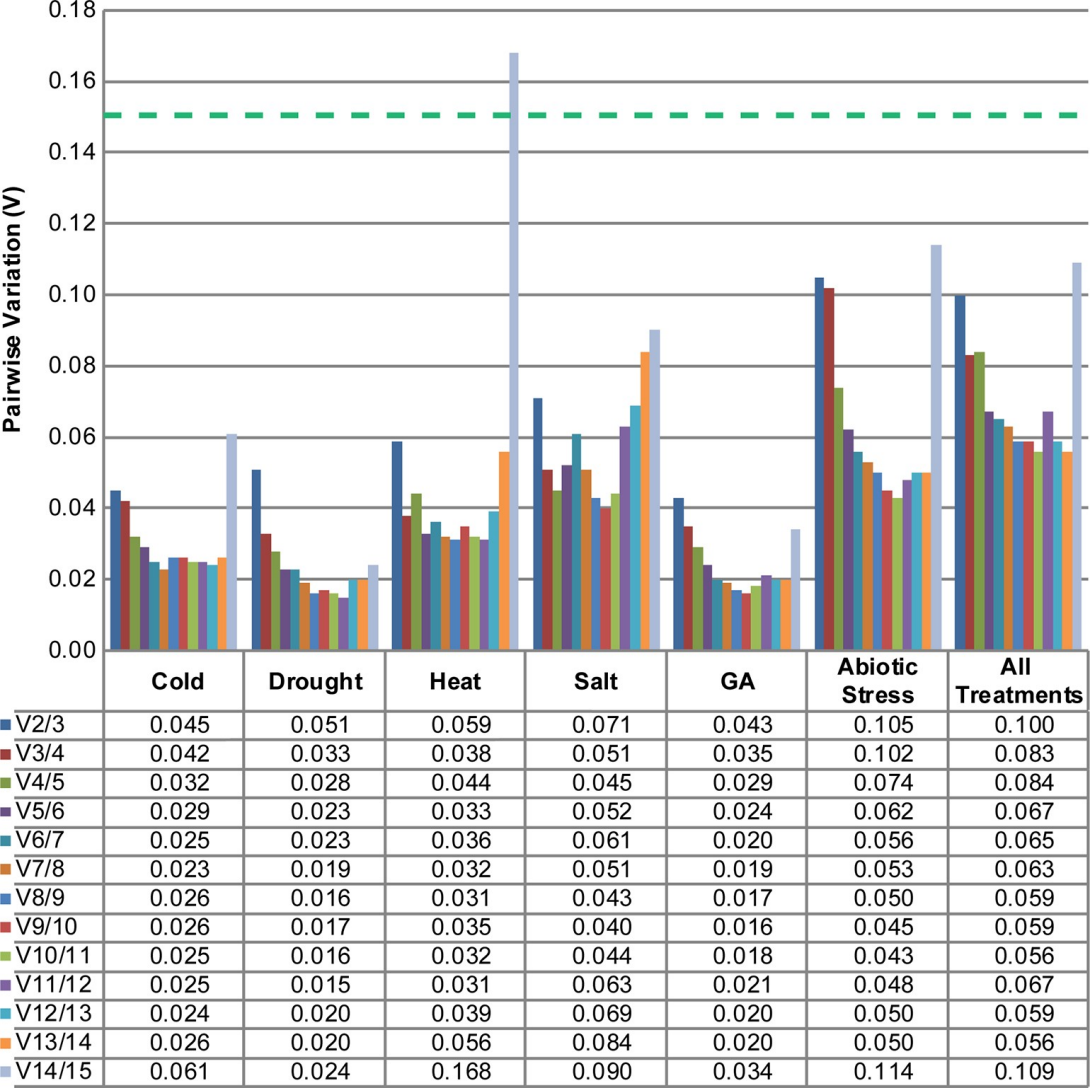
Optimal number of reference genes for various conditions. The geNorm algorithm was used to determine the pairwise variation (V) between the reference genes for treatments with cold, drought, heat, salt and gibberellic acid. The threshold for adequate normalisation is V≤0.15, indicated by the green dashed line.

For the optimal normalisation, it is necessary to use two or more reference genes in each experiment. The optimal number of reference genes can be determined with the geNorm algorithm, which calculates the pairwise variation V_n/n+1_ based on the normalisation factors NF_n_ and NF_n+1_, with n≥2. If V_n/n+1_ is below 0.15, n is the optimal number of reference genes. For all tested treatments, individually or combined, two reference genes are sufficient to normalise RT-qPCR measurements (Fig 2). The use of a third reference would not improve the results significantly.

### Impact of reference genes on the normalisation of samples in abiotic stress and hormone treatments

The efficiencies of treatments with cold, drought, heat, salt and gibberellic acid were determined by the expression analysis of specific stress response genes by RT-qPCR using primers for *RD29A* (*RESPONSIVE TO DESICCATION 29A*, cold and drought responsive), *HSP81*.*2/90* (heat responsive), *TSPO* (*OUTER MEMBRANE TRYPTOPHAN-RICH SENSORY PROTEIN-RELATED*, salt responsive) and *GA3ox1* (*GIBBERELLIN 3-OXIDASE 1*, GA responsive). All primers showed efficiencies of 80.28 to 104.22% and correlations of more than 0.99 (Table 4).

**Table 4:** Abiotic stress and hormone responsive genes, primers and amplicons.

Name	Gene ID	Arabidopsis homolog	Primer sequence (5’ to 3’)		Amplicon length	E [%]	R^2^
*RD29A*	Aa_G396870	AT5G52310	F R	GCCCTTGCTTCAGGGCTAGG TGCTCCGGTGTTTCCACTCC	89 bp	80.28	-0.996
*HSP81*.*2/90*	NA	AT5G56030	F R	CGTCTGGTGAGGCTCTTGGT AGCCTACGCTCCTCAAGGTACT	85 bp	96.74	-0.999
*TSPO*	Aa_G12840	AT2G47770	F R	GTGGACGGTGGGTTCCACAA CACACCACAAGCCCGGCTAA	125 bp	104.22	-0.994
*GA3ox1*	Aa_G112460	AT1G15550	F R	TTCCGGTTACCTGTCCAACG GCCTGAGATGGTGAAGCCTT	129 bp	82.79	-0.999

NA–not annotated (identified by nucleotide BLAST), F–forward primer, R–reverse primer, E[%]–efficiency of the primers was calculated with $E[\%]=100\times {(10}^{\left(-\frac{1}{slope}\right)}-1)$, slope–average Cq values (y) and log10 values of six serial dilutions (x) were used to calculate the slope of a regression line with the formula $slope=\frac{\sum \left(x-\bar{x})(y-\bar{y}\right)}{{\sum \left(x-\bar{x}\right)}^{2}}$, R^2^ –correlation of the Cq values calculated with the formula $\rho_{x,y}=\frac{Cov(X,Y)}{\sigma_{x}\times \sigma_{y}}$.

**Fig 3:**
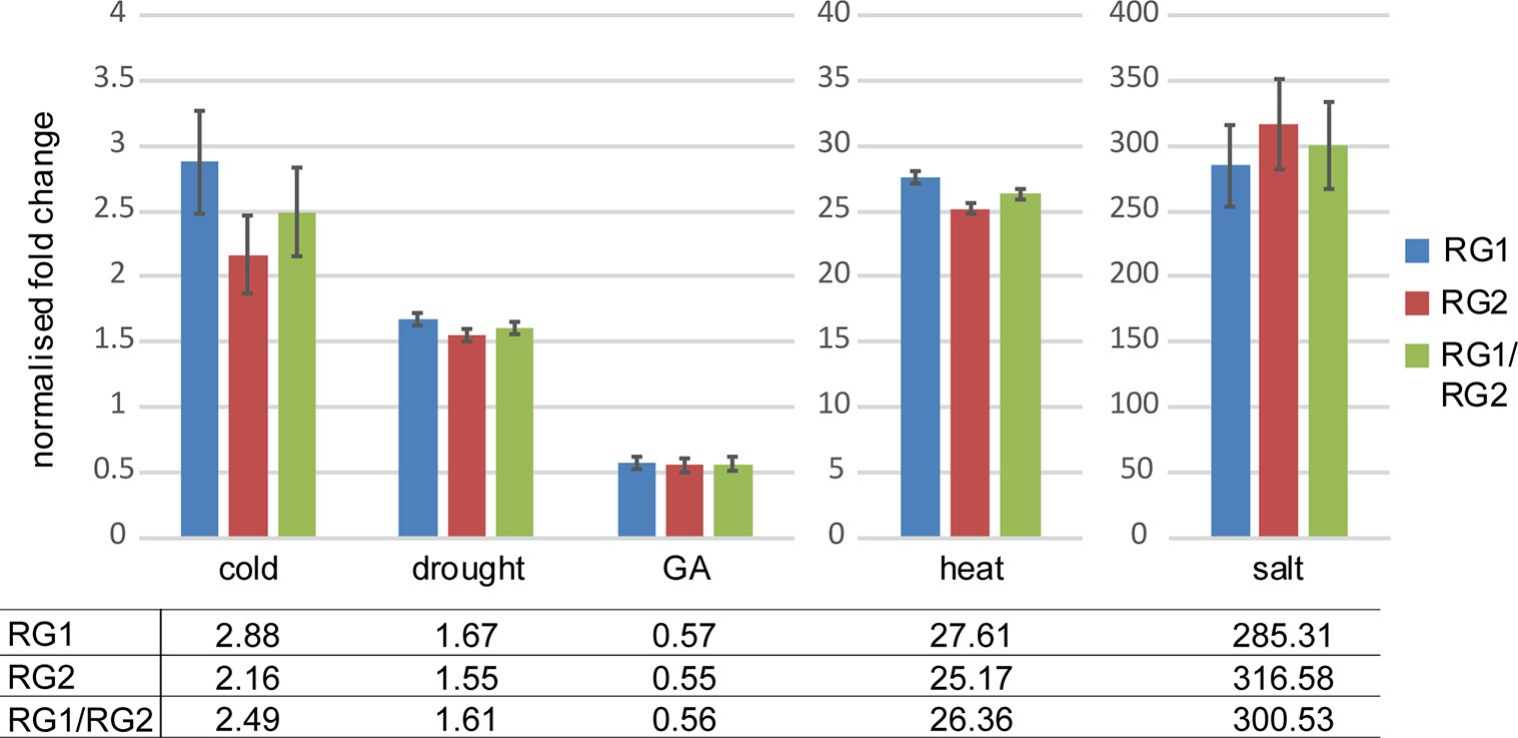
Comparison of specific stress response genes normalised with different reference genes. Normalisation of the stress response genes RD29A (RESPONSIVE TO DESICCATION 29A, cold and drought responsive), HSP81.2/90 (heat responsive), TSPO (OUTER MEMBRANE TRYPTOPHAN-RICH SENSORY PROTEIN-RELATED, salt responsive) and GA3ox1 (GIBBERELLIN 3-OXIDASE 1, GA responsive) was carried out with one or two reference genes (RG1 and RG2): cold–RAN3 and HCF, drought–UBQ10 and TUA5, GA and salt–PSB33 and TUA5, heat–RAN3 and PSB33.

Normalisation of the results was carried out with one or two of the most stable reference genes determined in this study (Fig 3). For cold, normalisation was carried out with *RAN3* and/or *HCF*. Drought samples were normalised to *UBQ10* and/or *TUA5*. G*A* and salt responsive genes were normalised with *PSB33* and/or *TUA5*. Finally, the heat stress samples were normalised with *RAN3* and/or *PSB33*. The results clearly show that all treatments were successful, leading to an increased (cold, drought, heat, salt) or decreased (GA) expression of the responsive genes. Individual normalisation with each reference gene led to differences in the calculated fold changes of 28.9% (cold), 7.5% (drought), 3.6% (GA), 9.3% (heat) and 10.4% (salt) between reference gene 1 and 2, respectively. These results clearly show the necessity to use two reference genes simultaneously.

## Material and methods

### Plant growth conditions

For the abiotic stress treatments, seeds were surface sterilised with increasing concentrations of ethanol and grown on MS [11] plates without additional sucrose. The seeds were stratified for five days. Subsequently, plants were grown under long day conditions (16h light/8h darkness) at 21±1°C and 100±20 μmol/m^2^s light intensity.

The heat and cold treatments were carried out by transferring 5–7 day old seedlings to 38°C and 4°C, respectively. Samples were taken after 2 h. Drought was induced on MS plates which were exposed to liquid MS containing 20% PEG 8000 for 24 h prior to the experiment. Samples were taken after 24 h. For salt treatments, two to three 5–7 day old seedlings were transferred from plates to liquid ½ MS (control) or liquid ½ MS containing 125 mM NaCl for 4 h under constant shaking.

For the GA treatment, plants were grown on soil under long day conditions (16h light/8h darkness) at 21±1°C and 200±20 μmol/m^2^s light intensity. The treatment was started right after germination and continued twice per week. Plants were sprayed with 20 μM GA4 (Sigma Aldrich, stock solution: 100 mM GA4 in EtOH, 0.1% Silwet L-77 Loveland industries) or mock (0.1% EtOH, 0.1% Silwet). Leaves were harvested 14 days after germination at Zeitgeber time (ZT) 8.

All experiments were performed in three independent biological replicates. All samples were immediately frozen in liquid nitrogen and stored at -80°C.

### RNA extraction and cDNA synthesis

The frozen seedling samples were ruptured using a TissueLyser (Qiagen) and total RNA extraction was carried out with Tri-Reagent (Ambion by Life Technologies). All samples were treated with DNAseI (Thermo Fisher Scientific). The frozen leaf samples were ruptured using a TissueLyser (Qiagen), extracted with the RNAeasy Plant Mini Kit (Qiagen) and treated with DNAse (Ambion by Life Technologies). RNA was analysed on a bleach gel [12], resulting in distinct bands for 28s and 18s rRNA, respectively. RNA concentration and purity was measured using a photometer (Eppendorf). cDNA was synthesised from 500 ng total RNA with oligodT primers, using the RevertAid First Strand cDNA Synthesis Kit (Thermo Fisher Scientific) according to the provided protocol. The cDNA was tested for DNA contamination and integrity by PCR and subsequent gel electrophoresis. The cDNA was diluted (1:10, 1:20, 1:40, 1:80, 1:160, and 1:320) to analyse primer efficiency and determine the correlation coefficient.

### Choice of candidate genes and primer design

The eight reference gene candidates *EIF4a*, *ACTIN 2*, *CAC*, *TUA5*, *HISTONE H3*, *HSP81*.*2/90*, *18srRNA* and *SAND* were chosen due to their stable expression in other species [13–17]. *PSB33*, *ATPase*, *THIOREDOXIN*, *HCF* and *NdhO* were chosen because of their robust expression levels in *Arabis alpina* in a time-course RNAseq experiment (data provided by Eva Willing, MPIPZ, Cologne) and/or due to their essential function for the plant. The *RAN3* primers were taken from[1]. *UBQ10* primers were kindly provided by Pan Pan Jiang (Botanical Institute, University of Cologne).

Sequences were taken from TAIR [18], NCBI (National Centre for Biotechnology Information, www.ncbi.nlm.nih.gov) and the Genomic resources for *Arabis alpina* website (www.arabis-alpina.org) [2]. *In silico* sequence analysis was carried out with CLC DNA Workbench version 5.6.1. The primers were designed using GenScript Real-time PCR Primer Design (www.genscript.com) at an optimum Tm of 60±2°C. Amplicons showed a single band of the expected size in gel electrophoresis and a single peak in the melting curve (S1 Fig). The PCR products were sequenced by GATC/ Eurofins Genomics to confirm specific amplification, using published sequences as a reference (www.arabis-alpina.org) [2].

### Quantitative real-time PCR (qPCR)

qPCRs were carried out in a QuantStudio 5 System (ABI/Life Technologies) equipped with the QuantStudio Design and Analysis Software version 1.4.1. The qPCRs were performed using plates (96 well, 0.2 ml) and cover foil (Opti-Seal Optical Disposable Adhesive) from BIOplastics. Reaction mixtures of 10 μl were composed from 5 μl SYBR Green (Thermo Fisher Scientific), 0.2 μl of each primer (10 μM), 1 μl diluted cDNA and 3.6 μl ddH_2_O. Amplification was carried out with the standard settings of the QuantStudio 5 System (50°C for 2 min, 95°C for 10 sec, 40 cycles at 95°C for 15 sec and 60°C for 1 min, followed by 95°C for 15 sec and a final dissociation curve from 60°C to 95°C). For each reference gene sample, three biological and three technical replicates were analysed. The impact of normalisation on stress/hormone responsive genes was analysed for 1:40 diluted cDNA in three technical replicates of one biological replicate.

### Analysis of RT-qPCR data

Efficiency calculations were carried out manually using Excel 2007. Outliers were removed using a two-sided Grubbs test at a significance level of 0.05. Efficiency of primers was calculated using the cDNA dilution series. The average Cq values (y) and log10 values of the dilutions (x) were used to calculate the slope of a regression line with the formula $slope=\frac{\sum \left(x-\bar{x})(y-\bar{y}\right)}{{\sum \left(x-\bar{x}\right)}^{2}}$. The slope was then used to calculate the efficiency of the primers $E[\%]=100\times {(10}^{\left(-\frac{1}{slope}\right)}-1)$. The correlation R^2^ of the values was calculated using the formula $\rho_{x,y}=\frac{Cov(X,Y)}{\sigma_{x}\times \sigma_{y}}$. Primers for reference genes were accepted with an efficiency of 90–110% and a correlation between -1 and -0.99. Primers for genes of interest were accepted with an efficiency of 80–120% and a correlation between -1 and -0.99.

The stability of the reference genes within a given set of different treatments was calculated from the efficiency corrected data using the algorithms NormFinder [8], BestKeeper [9] and geNorm [10]. The geNorm algorithm was also used to define the number of reference genes necessary for normalisation.

Normalisation against one reference gene was carried out using the normalisation factor, normalisation against two reference genes was carried out using the geometric mean of the normalisation factors, according to the geNorm manual [10]. In accordance with this manual, standard deviations between biological replicates were calculated over the means of the single replicates, rather than the raw data.

## Discussion

Gene expression analysis by RT-qPCR is a high-throughput method, which is considered to be very sensitive and reproducible. However, the accuracy of the results strongly depends on the experimental design, adequate normalisation and exact analysis of the produced data [7]. Moreover, the RT-qPCR primers must be specific, efficient, and—in the case of reference genes—stable in the tested conditions [7]. In this study, we analysed primers for one established reference gene (*RAN3*) [1] and 14 novel reference genes for *Arabis alpina* in several abiotic stress and hormone treatments. *EIF4a*, *ACTIN 2*, *CAC*, *TUA5*, *HISTONE H3*, *HSP81*.*2/90* and *SAND* were chosen because they were already established as reference genes in other species [13–17,19,20]. In addition, we considered *PSB33*, *ATPase*, *THIOREDOXIN*, *HCF* and *NdhO*. The reference genes selected here are involved in various basic cellular functions including translation, proton transport, photosynthesis, protein degradation and cytoskeletal organisation. Our data suggest that all genes reported here are appropriate reference genes for the tissues used under non-stress conditions. As *PSB33* and *HCF* are functionally related to photosynthesis, they may be more appropriate for photosynthetic tissues.

The reference gene primers presented here have efficiency values between 96.42 and 107.01%. Although these are very good efficiencies, it is essential to correct the RT-qPCR results with these values because of the non-linearity of the PCR amplification steps [21]. Consequently, we used efficiency-corrected data for identifying the most stable reference genes and for the analysis of the impact on normalisation. There is currently no consensus in the community, which of the three statistical algorithms–geNorm, NormFinder or BestKeeper–is most appropriate. One advantage of the geNorm algorithm is that it can be used for small sample sizes [22]. However, the geNorm method is biased towards genes that are co-regulated [23]. The algorithm takes into consideration, whether genes show a similar expression pattern [24], since it is assumed that similar changes in the expression of two independent genes reflect technical differences, such as the cDNA concentration, rather than changes caused by a treatment. By contrast, NormFinder considers variations across subgroups [8]. The algorithm assumes that there is no systematic variation of the average of the tested samples, which can lead to a preference for reference genes with similar systematic variation [23]. BestKeeper takes the standard deviation of each individual reference gene into account, which is an advantage over the other methods. The disadvantage of this algorithm is the use of a parametric method (Pearson correlation), which requires normally distributed data with a homogenous variance [9], which is not always the case. Additionally, BestKeeper uses the raw Cq values, while geNorm and Normfinder require normalised quantities. Therefore, the results obtained with BestKeeper are often different from those of the other two methods.

It is generally recommended to use more than one reference gene to guarantee optimal normalisation [7]. Our data support this view. The normalised fold change values varied up to 28.9% between two references. Moreover, we recommend that at least one of the reference gene amplicons contains introns at the genomic level to recognize potential contaminations with genomic DNA.

For cold treatments, we recommend *RAN3*, as the amplicon contains an intron, combined with *HCF* or *PSB33*. As *HCF* and *PSB33* are photosynthesis-associated proteins located in the thylakoid membrane, there is no obvious reason to prefer one over the other. For drought treatments, *UBQ10* and *TUA5* were the most stable transcripts, with *TUA5* also containing an intron. In heat, *RAN3* can be recommended in combination with *PSB33* or *EIF4a*. As setups for heat treatments often go along with specific light settings, it might be advisable to use *EIF4a* as a second reference here. For the salt treatment, we found that *CAC*, *TUA5* and *ACTIN 2* are suitable, intron containing reference genes, which can be combined with each other or *PSB33*. The combination *TUA5* and *PSB33* might be the most efficient choice, as it is also the best option for GA treatments.

We found that there is no single reference gene, which is the best choice for all treatments. However, all genes tested in this study, except for *HSP81*.*2/90* under heat conditions, meet the requirements necessary for adequate normalisation. Thus, this study provides data for the selection of suitable reference gene combinations for *Arabis alpina* under cold, drought, heat, salt and GA treatments. With this, we enable adequate normalisation in RT-qPCR experiments under these conditions and provide novel reference genes for future experiments addressing other stresses or stimuli.

## Supporting information

S1 Table Raw Cq values of treatments with cold, drought, heat, salt and gibberellic acid. (DOCX)

S1 Fig Amplicon analysis of candidate reference genes and stress/hormone responsive genes. (DOCX)

S2 Fig Standard curves of candidate reference genes and stress/hormone responsive genes. (DOCX)

S2 Table Ranking of gene expression stability under abiotic stress conditions and the combination of all treatments. (DOCX)
