# Supplementary material for: Selection and validation of reference genes for quantitative Real-Time PCR in Arabis alpina
Source: PLoS One. 2019 Mar 4;14(3):e0211172. doi: 10.1371/journal.pone.0211172 (PMC6398851; doi:10.1371/journal.pone.0211172)
Supplement: S1 Table — (DOCX) [file pone.0211172.s001.docx]

**S1 Table.** **Raw Cq values of treatments with cold, drought, heat, salt and gibberellic acid.**

| sample | replicate | | ATPase | PSB33 | THIOREDOXIN | HISTONE H3 | HCF | NdhO | EIF4a | TUA5 | RAN3 | 18srRNA | UBQ10 | CAC | ACTIN 2 | SAND | HSP81.2/90 |
| --- | --- | --- | --- | --- | --- | --- | --- | --- | --- | --- | --- | --- | --- | --- | --- | --- | --- |
| salt control | T1 | B1 | 26.90 | 26.96 | 26.17 | 26.24 | 28.23 | 28.34 | 26.95 | 25.33 | 26.65 | 16.34 | 21.23 | 27.85 | 23.45 | 27.88 | 28.41 |
|  |  | B2 | 24.62 | 26.62 | 25.31 | 25.48 | 27.71 | 27.22 | 23.11 | 24.91 | 23.14 | 16.81 | 20.83 | 27.10 | 22.90 | 27.42 | 27.67 |
|  |  | B3 | 25.74 | 27.32 | 25.97 | 25.57 | 28.98 | 27.31 | 25.09 | 25.66 | 23.18 | 16.42 | 21.13 | 27.61 | 23.60 | 27.77 | 28.35 |
|  | T2 | B1 | 27.29 | 27.20 | 26.18 | 26.27 | 28.44 | 27.97 | 27.37 | 25.34 | 26.42 | 16.25 | 21.41 | 27.65 | 23.63 | 27.71 | 28.19 |
|  |  | B2 | 24.57 | 26.42 | 25.39 | 25.29 | 27.35 | 27.15 | 23.15 | 25.04 | 23.19 | 16.65 | 20.84 | 27.12 | 22.92 | 27.82 | 27.72 |
|  |  | B3 | 25.94 | 26.94 | 25.97 | 25.60 | 27.88 | 27.47 | 25.15 | 25.66 | 23.13 | 16.45 | 21.33 | 27.72 | 23.68 | 27.75 | 27.88 |
|  | T3 | B1 | 26.84 | 27.13 | 26.02 | 26.23 | 28.16 | 28.75 | 26.86 | 25.35 | 26.38 | 16.24 | 21.17 | 27.65 | 23.56 | 27.83 | 28.60 |
|  |  | B2 | 24.39 | 26.34 | 25.05 | 25.38 | 27.44 | 27.39 | 23.15 | 24.82 | 23.12 | 16.54 | 20.75 | 27.15 | 22.90 | 27.13 | 27.63 |
|  |  | B3 | 25.72 | 27.25 | 25.68 | 25.39 | 27.89 | 27.32 | 25.13 | 25.28 | 23.21 | 16.31 | 20.97 | 27.33 | 23.54 | 27.67 | 27.84 |
| salt | T1 | B1 | 26.94 | 27.23 | 25.86 | 25.48 | 27.56 | 27.42 | 26.46 | 25.39 | 26.40 | 16.02 | 20.83 | 27.78 | 23.97 | 28.55 | 26.19 |
|  |  | B2 | 26.53 | 26.58 | 25.58 | 25.11 | 27.21 | 27.10 | 25.95 | 25.28 | 25.69 | 15.47 | 20.14 | 27.25 | 23.38 | 28.20 | 26.42 |
|  |  | B3 | 25.92 | 26.92 | 25.37 | 25.13 | 27.14 | 27.17 | 25.92 | 25.10 | 25.65 | 15.97 | 20.07 | 27.15 | 23.53 | 28.14 | 26.10 |
|  | T2 | B1 | 26.91 | 27.10 | 26.19 | 25.35 | 27.78 | 27.96 | 26.30 | 25.38 | 26.27 | 16.09 | 20.87 | 27.82 | 23.89 | 28.39 | 25.97 |
|  |  | B2 | 26.47 | 26.50 | 25.86 | 24.80 | 26.88 | 27.12 | 25.91 | 24.96 | 26.60 | 15.63 | 20.15 | 27.60 | 23.35 | 27.64 | 26.35 |
|  |  | B3 | 26.12 | 26.75 | 26.17 | 24.94 | 26.76 | 27.54 | 26.00 | 24.84 | 25.51 | 15.98 | 19.96 | 27.30 | 23.41 | 27.62 | 26.31 |
|  | T3 | B1 | 26.92 | 27.45 | 26.27 | 25.36 | 27.31 | 27.35 | 26.23 | 25.43 | 26.12 | 16.21 | 20.80 | 27.76 | 23.96 | 28.50 | 25.99 |
|  |  | B2 | 26.43 | 26.52 | 25.63 | 24.73 | 27.15 | 27.37 | 25.82 | 25.34 | 25.90 | 15.92 | 20.08 | 27.64 | 23.16 | 27.53 | 26.18 |
|  |  | B3 | 26.11 | 26.73 | 25.41 | 25.19 | 26.61 | 27.55 | 26.16 | 24.99 | 25.44 | 16.21 | 20.11 | 27.39 | 23.38 | 28.20 | 26.10 |
| GA control | T1 | B1 | 23.29 | 23.46 | 22.58 | 23.33 | 24.46 | 23.87 | 23.01 | 22.81 | 23.73 | 16.13 | 20.21 | 25.40 | 21.78 | 25.95 | 25.21 |
|  |  | B2 | 22.87 | 23.01 | 22.27 | 23.21 | 23.98 | 23.08 | 22.89 | 22.36 | 23.82 | 14.71 | 19.40 | 25.05 | 21.39 | 25.68 | 24.34 |
|  |  | B3 | 23.10 | 23.41 | 22.08 | 23.49 | 24.12 | 23.60 | 22.82 | 22.43 | 23.72 | 14.44 | 19.23 | 24.97 | 21.52 | 25.71 | 24.88 |
|  | T2 | B1 | 23.32 | 23.47 | 22.74 | 23.51 | 24.34 | 23.82 | 22.91 | 22.76 | 23.87 | 16.27 | 20.20 | 25.50 | 21.83 | 26.02 | 25.41 |
|  |  | B2 | 22.88 | 23.08 | 22.26 | 23.18 | 23.62 | 23.28 | 22.90 | 22.28 | 23.77 | 14.65 | 19.35 | 24.96 | 21.43 | 25.68 | 24.55 |
|  |  | B3 | 23.24 | 23.27 | 22.24 | 22.94 | 24.25 | 23.59 | 23.09 | 22.27 | 23.48 | 14.74 | 19.74 | 24.95 | 21.66 | 25.65 | 24.88 |
|  | T3 | B1 | 23.31 | 23.36 | 22.60 | 23.49 | 24.53 | 23.76 | 23.00 | 22.77 | 23.81 | 16.27 | 20.14 | 25.57 | 21.72 | 26.06 | 25.28 |
|  |  | B2 | 22.72 | 23.20 | 22.01 | 23.14 | 23.82 | 23.30 | 22.82 | 22.34 | 23.68 | 14.69 | 18.99 | 25.07 | 21.34 | 25.86 | 24.47 |
|  |  | B3 | 23.42 | 23.26 | 22.38 | 22.96 | 24.29 | 23.51 | 22.90 | 22.50 | 23.59 | 14.71 | 19.36 | 25.06 | 21.61 | 25.78 | 24.70 |
| GA | T1 | B1 | 23.02 | 23.58 | 22.54 | 23.93 | 24.24 | 23.52 | 23.28 | 22.66 | 23.90 | 14.75 | 20.10 | 25.51 | 21.62 | 26.13 | 25.39 |
|  |  | B2 | 22.36 | 22.87 | 21.81 | 22.41 | 23.78 | 23.11 | 22.61 | 22.04 | 23.32 | 14.60 | 19.23 | 24.74 | 21.20 | 25.66 | 24.17 |
|  |  | B3 | 22.74 | 23.01 | 22.12 | 22.88 | 23.90 | 23.05 | 22.54 | 22.09 | 23.35 | 15.20 | 19.43 | 25.09 | 21.20 | 25.66 | 24.49 |
|  | T2 | B1 | 23.11 | 23.44 | 22.36 | 23.82 | 24.24 | 23.69 | 23.06 | 22.66 | 23.90 | 14.66 | 20.19 | 25.46 | 21.60 | 26.22 | 25.35 |
|  |  | B2 | 22.13 | 22.99 | 21.64 | 22.51 | 23.70 | 22.94 | 22.41 | 22.08 | 23.25 | 14.74 | 19.18 | 24.85 | 21.14 | 25.48 | 23.98 |
|  |  | B3 | 22.71 | 22.95 | 22.00 | 22.97 | 24.08 | 23.16 | 22.54 | 22.31 | 23.48 | 15.18 | 19.32 | 25.08 | 21.16 | 25.59 | 24.64 |
|  | T3 | B1 | 23.08 | 23.58 | 22.52 | 23.86 | 24.26 | 23.51 | 23.14 | 22.76 | 23.99 | 14.78 | 20.02 | 25.50 | 21.65 | 26.18 | 25.40 |
|  |  | B2 | 22.36 | 22.89 | 21.72 | 22.48 | 23.60 | 23.15 | 22.34 | 22.21 | 23.16 | 14.85 | 19.11 | 24.84 | 21.07 | 25.91 | 23.85 |
|  |  | B3 | 22.82 | 23.07 | 22.08 | 23.15 | 23.90 | 23.12 | 22.44 | 22.34 | 23.51 | 15.32 | 19.33 | 25.13 | 20.94 | 25.57 | 24.57 |
| drought control | T1 | B1 | 24.36 | 24.83 | 24.31 | 23.00 | 25.86 | 25.22 | 23.77 | 23.91 | 23.82 | 15.44 | 20.05 | 26.36 | 22.83 | 26.74 | 24.31 |
|  |  | B2 | 23.91 | 24.60 | 23.60 | 22.41 | 25.61 | 24.96 | 23.24 | 23.24 | 22.95 | 14.25 | 19.50 | 25.61 | 22.27 | 26.03 | 23.85 |
|  |  | B3 | 24.37 | 24.99 | 24.01 | 22.80 | 25.71 | 24.96 | 23.70 | 23.61 | 23.43 | 14.84 | 19.86 | 26.07 | 22.68 | 26.43 | 24.33 |
|  | T2 | B1 | 24.28 | 25.02 | 24.41 | 23.23 | 25.81 | 25.44 | 23.79 | 23.76 | 23.72 | 15.62 | 19.99 | 26.26 | 22.81 | 26.80 | 24.39 |
|  |  | B2 | 24.10 | 24.73 | 23.72 | 22.50 | 25.63 | 25.13 | 23.14 | 23.26 | 22.90 | 14.39 | 19.68 | 25.55 | 22.24 | 25.88 | 23.90 |
|  |  | B3 | 24.18 | 24.94 | 23.98 | 22.79 | 25.77 | 25.12 | 23.64 | 23.60 | 23.80 | 14.77 | 19.79 | 26.16 | 22.58 | 26.25 | 24.48 |
|  | T3 | B1 | 24.32 | 24.92 | 24.19 | 23.01 | 25.94 | 25.29 | 23.71 | 23.76 | 23.71 | 15.54 | 19.88 | 25.96 | 22.87 | 26.34 | 24.50 |
|  |  | B2 | 23.84 | 24.68 | 23.68 | 22.44 | 25.48 | 24.95 | 23.14 | 23.19 | 23.08 | 14.39 | 19.55 | 25.62 | 22.27 | 26.01 | 23.95 |
|  |  | B3 | 23.91 | 24.86 | 23.99 | 22.91 | 25.80 | 25.15 | 23.59 | 23.52 | 23.41 | 14.70 | 19.72 | 25.98 | 22.40 | 26.39 | 24.32 |
| drought | T1 | B1 | 23.45 | 24.01 | 23.47 | 23.00 | 25.37 | 24.86 | 22.83 | 23.26 | 22.66 | 14.37 | 19.17 | 25.30 | 21.70 | 25.65 | 23.72 |
|  |  | B2 | 23.46 | 24.21 | 23.42 | 22.82 | 25.14 | 24.46 | 22.99 | 23.17 | 22.69 | 14.41 | 19.43 | 25.39 | 22.09 | 26.13 | 23.92 |
|  |  | B3 | 23.37 | 23.88 | 23.38 | 22.88 | 24.97 | 24.46 | 22.97 | 23.16 | 22.83 | 14.58 | 19.25 | 25.68 | 21.86 | 25.65 | 23.98 |
|  | T2 | B1 | 23.54 | 24.09 | 23.42 | 22.94 | 25.39 | 24.80 | 23.05 | 23.30 | 22.82 | 14.50 | 19.14 | 25.35 | 21.77 | 25.95 | 23.67 |
|  |  | B2 | 23.57 | 24.13 | 23.30 | 22.81 | 25.12 | 24.70 | 22.88 | 23.33 | 22.57 | 14.31 | 19.31 | 25.60 | 22.08 | 25.80 | 24.79 |
|  |  | B3 | 23.44 | 23.82 | 23.21 | 22.87 | 25.14 | 24.57 | 22.81 | 23.27 | 22.75 | 14.68 | 19.22 | 25.50 | 21.86 | 25.73 | 23.75 |
|  | T3 | B1 | 23.82 | 24.11 | 23.36 | 23.18 | 25.31 | 24.72 | 22.90 | 23.29 | 22.76 | 14.47 | 19.25 | 25.40 | 21.59 | 26.05 | 23.74 |
|  |  | B2 | 23.60 | 24.39 | 23.38 | 22.82 | 25.31 | 24.71 | 22.94 | 23.29 | 22.70 | 14.37 | 19.31 | 25.51 | 22.29 | 25.91 | 24.12 |
|  |  | B3 | 23.43 | 23.95 | 23.66 | 22.88 | 24.98 | 24.55 | 22.85 | 23.27 | 22.71 | 14.71 | 19.25 | 25.48 | 21.82 | 25.76 | 23.82 |
| cold and heat control | T1 | B1 | 24.46 | 24.93 | 24.17 | 23.77 | 26.24 | 25.80 | 23.74 | 23.80 | 23.64 | 14.90 | 20.21 | 26.20 | 22.07 | 26.64 | 25.63 |
|  |  | B2 | 24.13 | 24.55 | 23.54 | 22.95 | 25.63 | 25.47 | 23.35 | 23.15 | 23.30 | 13.77 | 19.84 | 25.46 | 21.74 | 25.98 | 24.88 |
|  |  | B3 | 24.08 | 24.43 | 23.78 | 23.14 | 25.72 | 25.02 | 23.71 | 23.37 | 23.60 | 14.34 | 19.80 | 25.56 | 21.81 | 26.10 | 25.37 |
|  | T2 | B1 | 24.56 | 25.11 | 24.13 | 23.65 | 26.19 | 25.91 | 23.76 | 23.68 | 23.83 | 14.93 | 20.26 | 26.18 | 22.15 | 26.51 | 25.66 |
|  |  | B2 | 23.91 | 24.47 | 23.58 | 23.00 | 25.63 | 25.60 | 23.41 | 23.12 | 23.41 | 13.63 | 19.62 | 25.59 | 21.73 | 26.11 | 25.54 |
|  |  | B3 | 23.96 | 24.47 | 23.78 | 23.43 | 25.85 | 25.25 | 23.43 | 23.33 | 23.64 | 14.46 | 19.91 | 25.71 | 21.87 | 26.08 | 25.23 |
|  | T3 | B1 | 24.72 | 25.13 | 24.29 | 23.56 | 26.15 | 25.80 | 23.85 | 23.62 | 23.70 | 14.96 | 20.28 | 26.16 | 22.27 | 26.69 | 25.57 |
|  |  | B2 | 23.98 | 24.46 | 23.51 | 22.92 | 25.70 | 25.47 | 23.32 | 23.22 | 23.45 | 13.97 | 19.79 | 25.59 | 21.70 | 26.21 | 24.94 |
|  |  | B3 | 23.96 | 24.77 | 23.79 | 23.34 | 25.69 | 25.00 | 23.44 | 23.26 | 23.45 | 14.50 | 19.84 | 25.66 | 21.80 | 26.13 | 25.29 |
| heat | T1 | B1 | 24.81 | 24.82 | 24.56 | 23.90 | 25.98 | 25.87 | 23.92 | 24.40 | 23.75 | 14.13 | 19.04 | 26.74 | 22.75 | 27.60 | 20.73 |
|  |  | B2 | 24.27 | 24.55 | 23.98 | 23.75 | 25.70 | 25.80 | 23.70 | 24.22 | 23.58 | 14.70 | 19.01 | 26.47 | 22.47 | 27.64 | 20.68 |
|  |  | B3 | 24.64 | 24.57 | 24.44 | 24.10 | 25.53 | 25.35 | 23.93 | 24.30 | 23.88 | 14.75 | 18.68 | 26.70 | 22.60 | 27.66 | 20.71 |
|  | T2 | B1 | 24.75 | 24.85 | 24.11 | 23.79 | 26.17 | 25.86 | 23.91 | 24.50 | 23.84 | 14.12 | 18.91 | 26.87 | 22.65 | 27.90 | 20.80 |
|  |  | B2 | 24.35 | 24.43 | 23.92 | 23.59 | 25.65 | 25.22 | 23.76 | 24.25 | 23.63 | 14.82 | 18.91 | 26.43 | 22.48 | 27.28 | 20.71 |
|  |  | B3 | 24.65 | 24.61 | 23.92 | 23.64 | 25.78 | 25.17 | 23.71 | 24.30 | 23.55 | 14.79 | 18.71 | 26.90 | 22.82 | 27.56 | 20.57 |
|  | T3 | B1 | 24.70 | 24.81 | 24.24 | 24.03 | 25.97 | 25.87 | 23.83 | 24.53 | 23.80 | 14.24 | 18.91 | 26.82 | 22.60 | 27.76 | 20.72 |
|  |  | B2 | 24.45 | 24.36 | 24.24 | 23.64 | 25.51 | 25.30 | 23.55 | 24.22 | 23.56 | 14.91 | 19.01 | 26.53 | 22.38 | 27.49 | 20.71 |
|  |  | B3 | 24.39 | 24.66 | 24.03 | 23.86 | 25.75 | 25.55 | 23.60 | 24.52 | 23.65 | 14.87 | 18.59 | 26.68 | 22.63 | 27.65 | 20.48 |
| cold | T1 | B1 | 24.84 | 25.07 | 24.49 | 23.20 | 25.97 | 25.64 | 23.81 | 23.91 | 23.81 | 15.19 | 19.67 | 26.40 | 22.81 | 26.78 | 23.80 |
|  |  | B2 | 24.15 | 24.46 | 24.21 | 22.90 | 25.74 | 25.17 | 23.18 | 23.52 | 23.48 | 14.88 | 19.39 | 26.01 | 22.29 | 26.49 | 23.76 |
|  |  | B3 | 24.56 | 24.94 | 24.19 | 22.99 | 25.69 | 25.42 | 23.51 | 23.85 | 23.70 | 15.25 | 19.57 | 26.33 | 22.67 | 26.54 | 23.73 |
|  | T2 | B1 | 24.99 | 25.17 | 24.64 | 23.27 | 26.09 | 25.59 | 23.55 | 23.81 | 23.86 | 15.37 | 19.81 | 26.33 | 22.68 | 26.51 | 23.79 |
|  |  | B2 | 24.09 | 24.74 | 23.97 | 22.90 | 25.61 | 25.23 | 23.11 | 23.66 | 23.41 | 14.92 | 19.31 | 26.06 | 22.25 | 26.45 | 23.76 |
|  |  | B3 | 24.33 | 24.95 | 24.31 | 22.87 | 25.73 | 25.33 | 23.51 | 23.90 | 23.68 | 15.24 | 19.62 | 26.19 | 22.74 | 26.56 | 23.76 |
|  | T3 | B1 | 24.56 | 25.14 | 24.53 | 23.33 | 26.09 | 25.39 | 23.55 | 23.93 | 24.04 | 15.12 | 19.77 | 26.59 | 22.73 | 27.42 | 23.77 |
|  |  | B2 | 24.15 | 24.66 | 24.06 | 22.96 | 25.47 | 25.16 | 23.08 | 23.68 | 23.46 | 14.97 | 19.29 | 26.13 | 22.22 | 26.34 | 23.72 |
|  |  | B3 | 24.43 | 24.95 | 24.10 | 22.94 | 25.89 | 25.27 | 23.65 | 23.81 | 23.75 | 15.16 | 19.50 | 26.34 | 22.59 | 26.54 | 23.72 |
