# Supplementary material for: Selection and validation of reference genes for quantitative Real-Time PCR in Arabis alpina
Source: PLoS One. 2019 Mar 4;14(3):e0211172. doi: 10.1371/journal.pone.0211172 (PMC6398851; doi:10.1371/journal.pone.0211172)
Supplement: S1 Fig — (DOCX) [file pone.0211172.s002.docx]

| ***A*** | 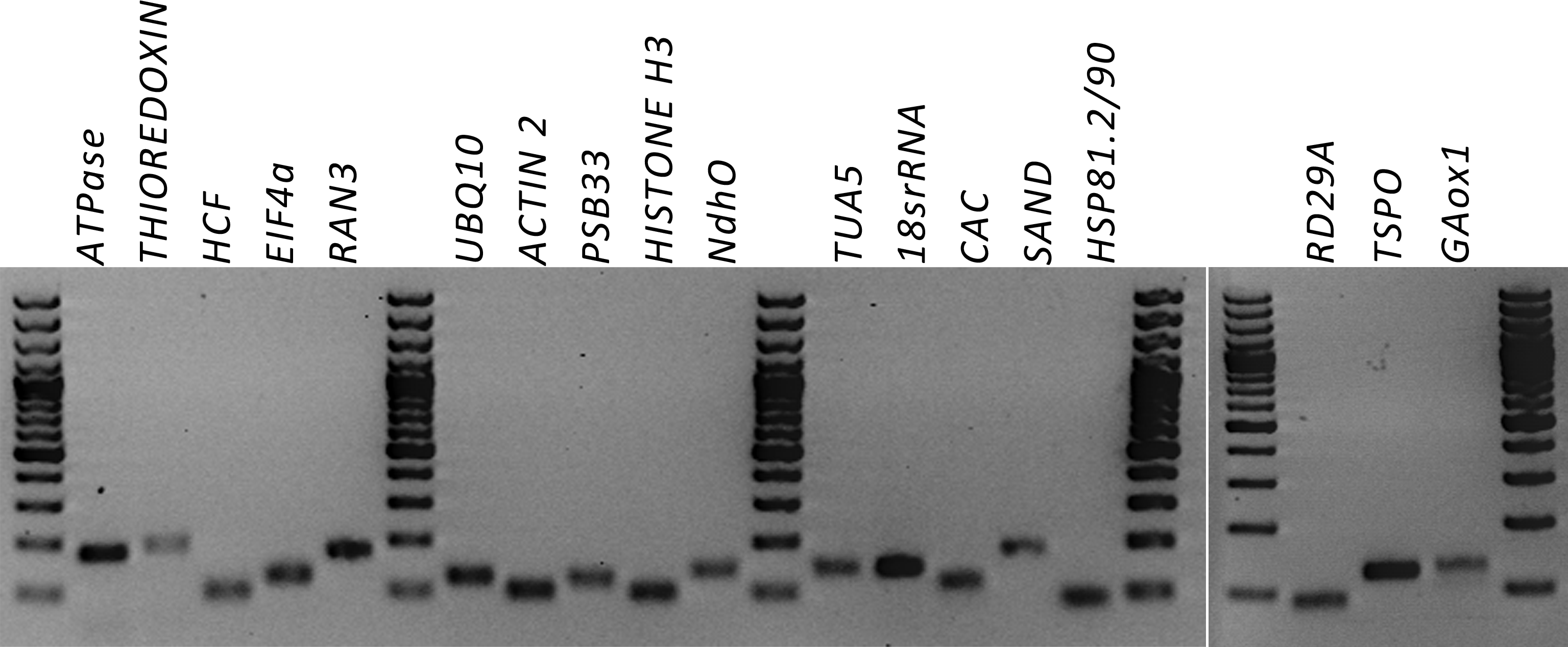 | |
| --- | --- | --- |
|  |  |  |
| ***B*** | ***ATPase 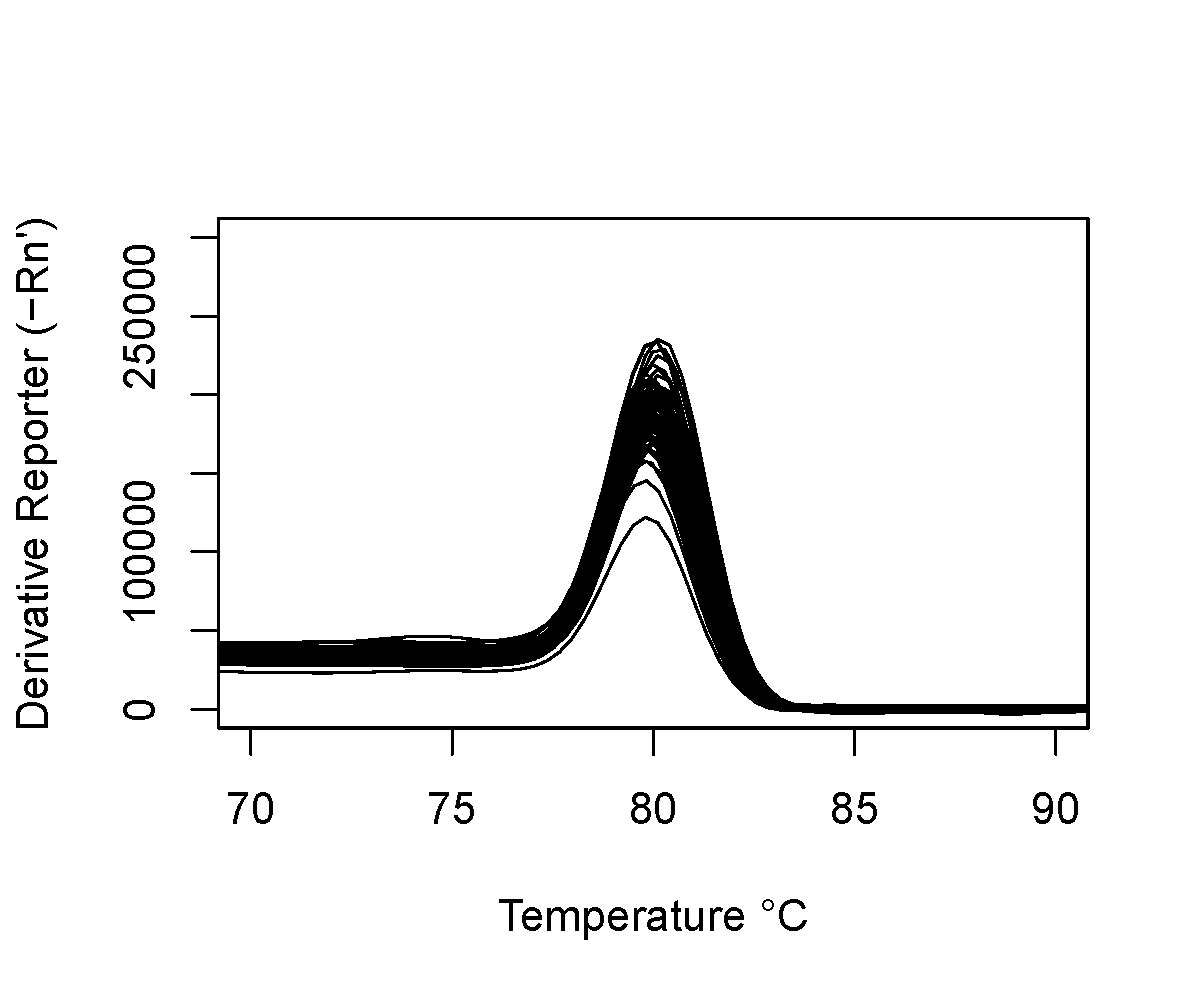*** | ***THIOREDOXIN 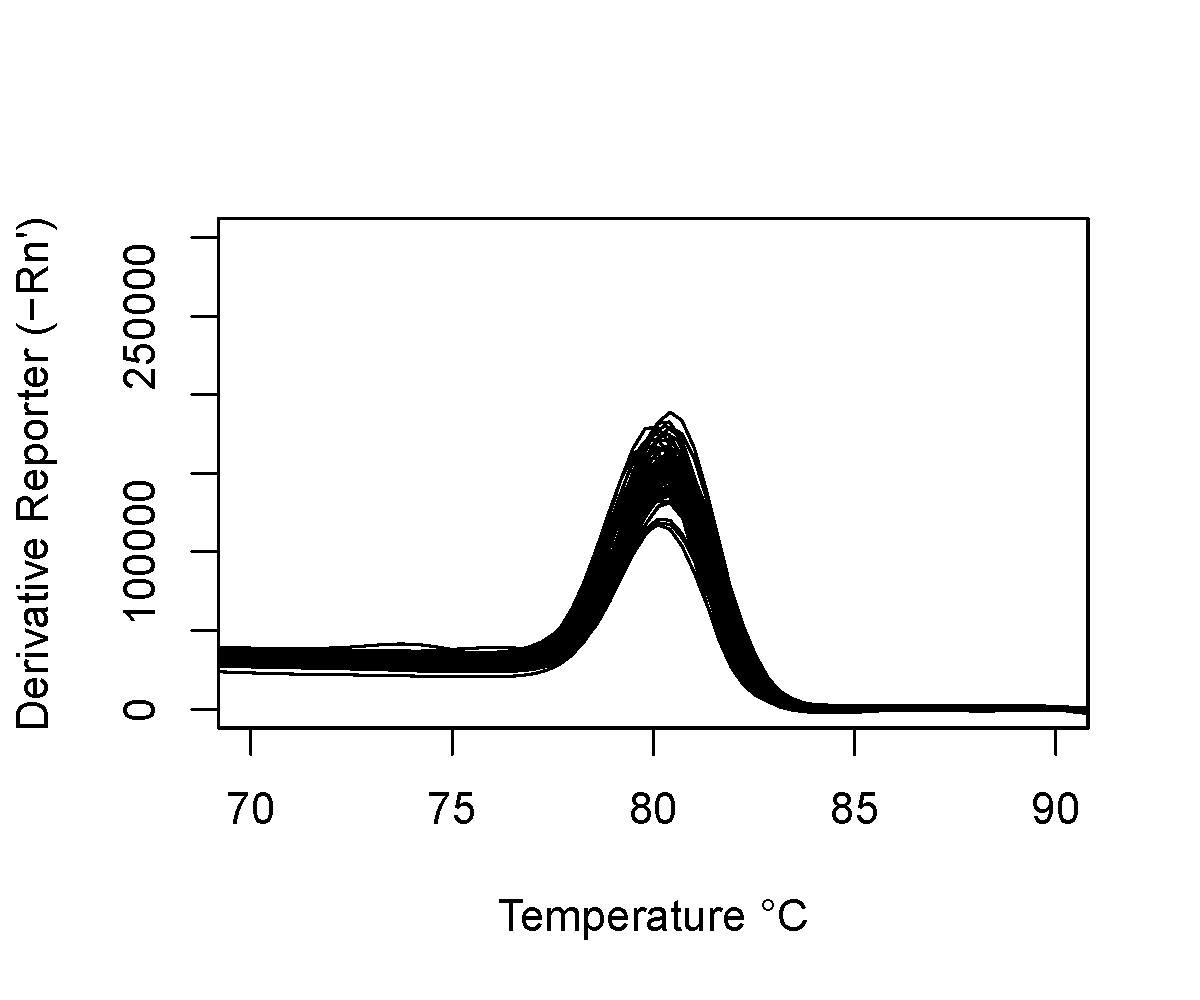*** |
|  | ***HCF 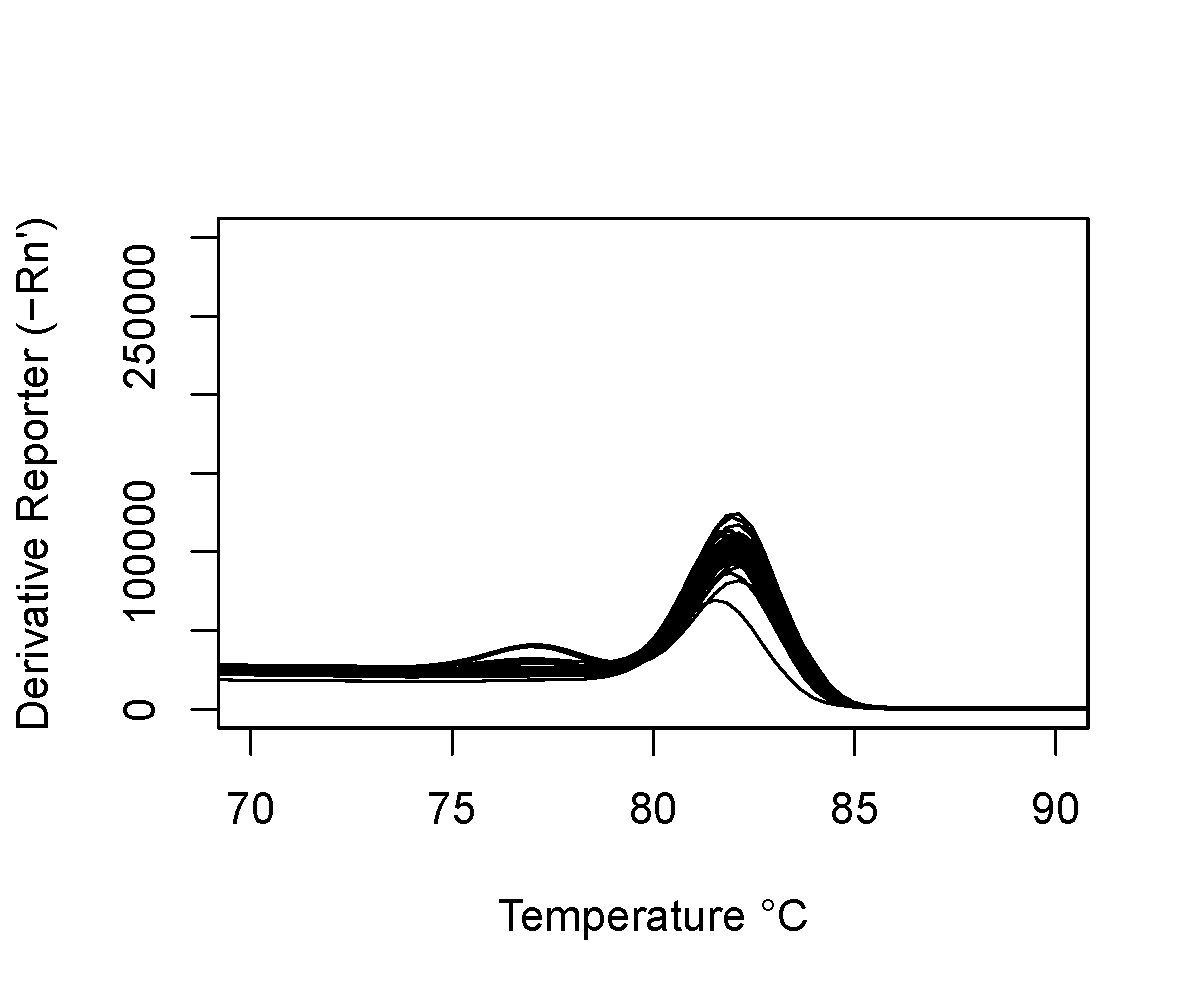*** | ***EIF4a 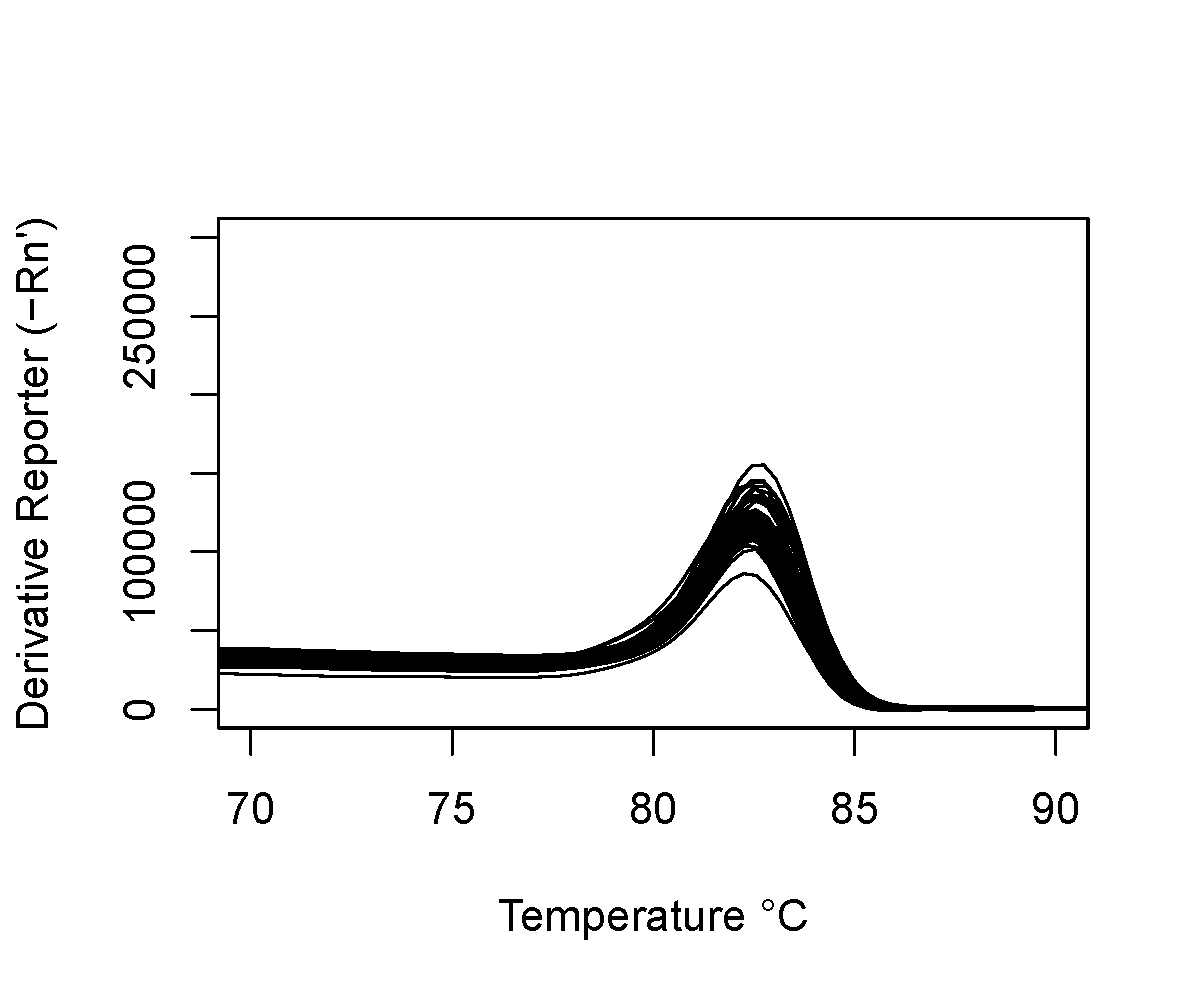*** |
|  | ***RAN3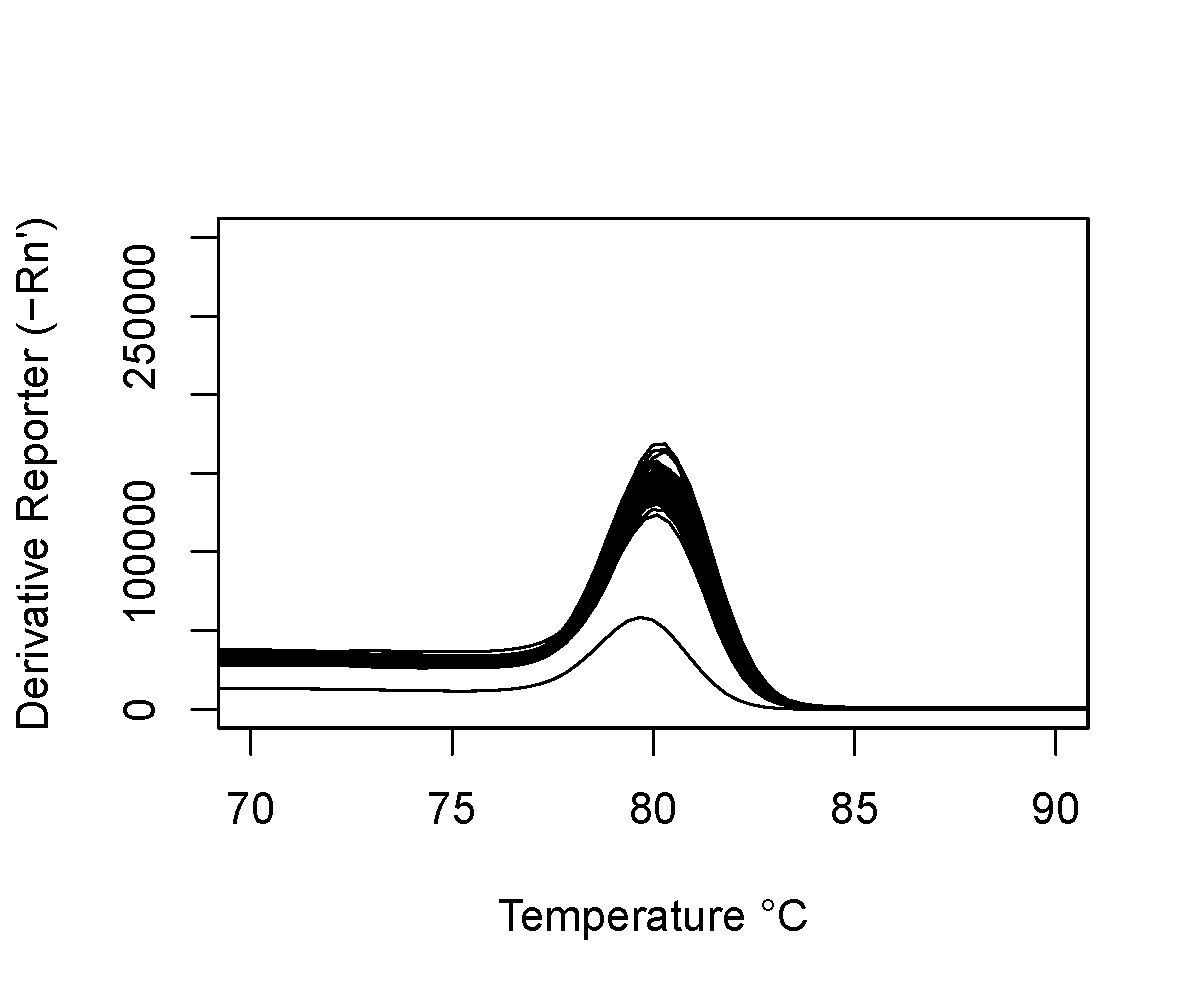*** | ***UBQ10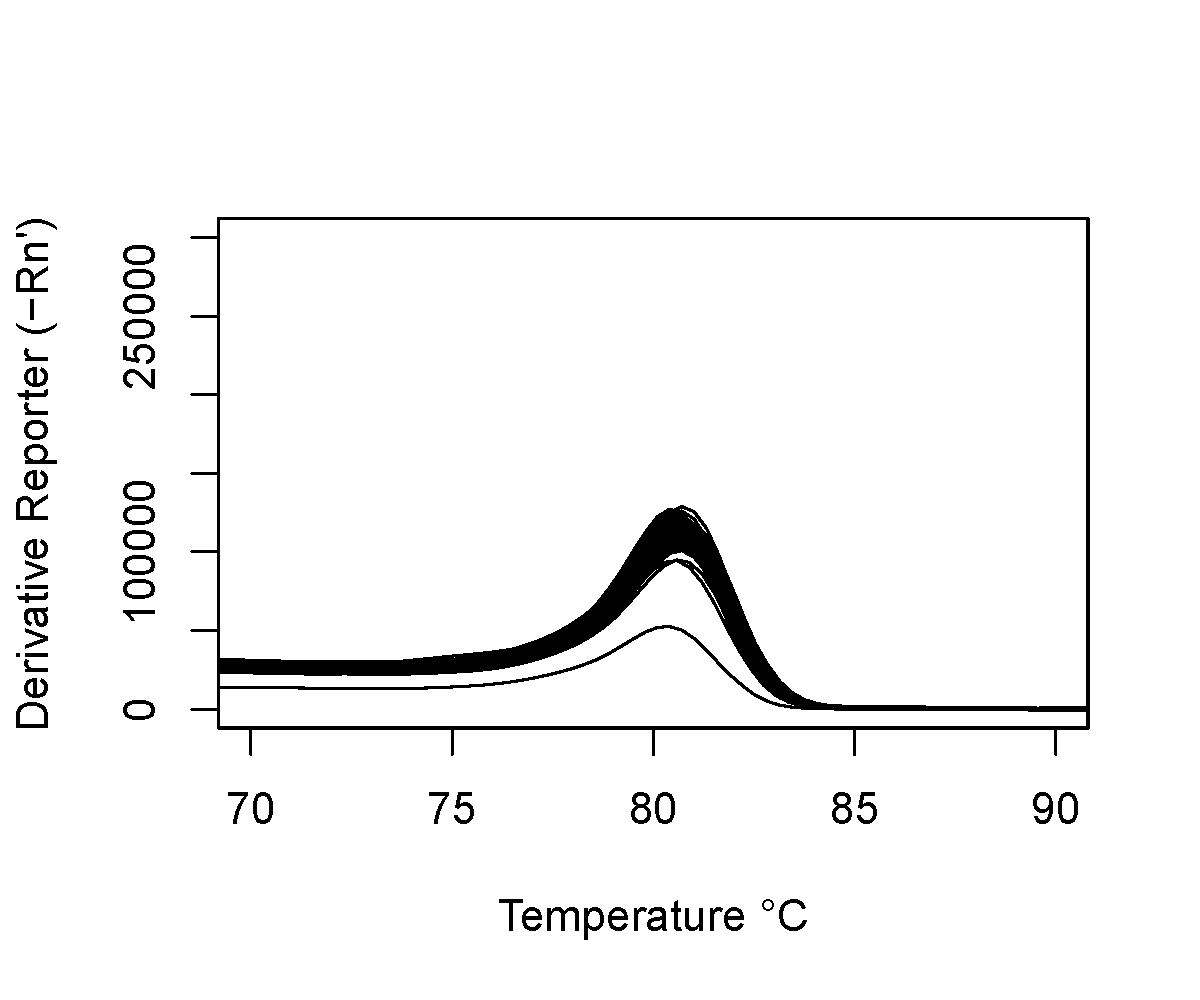*** |
|  | ***ACTIN 2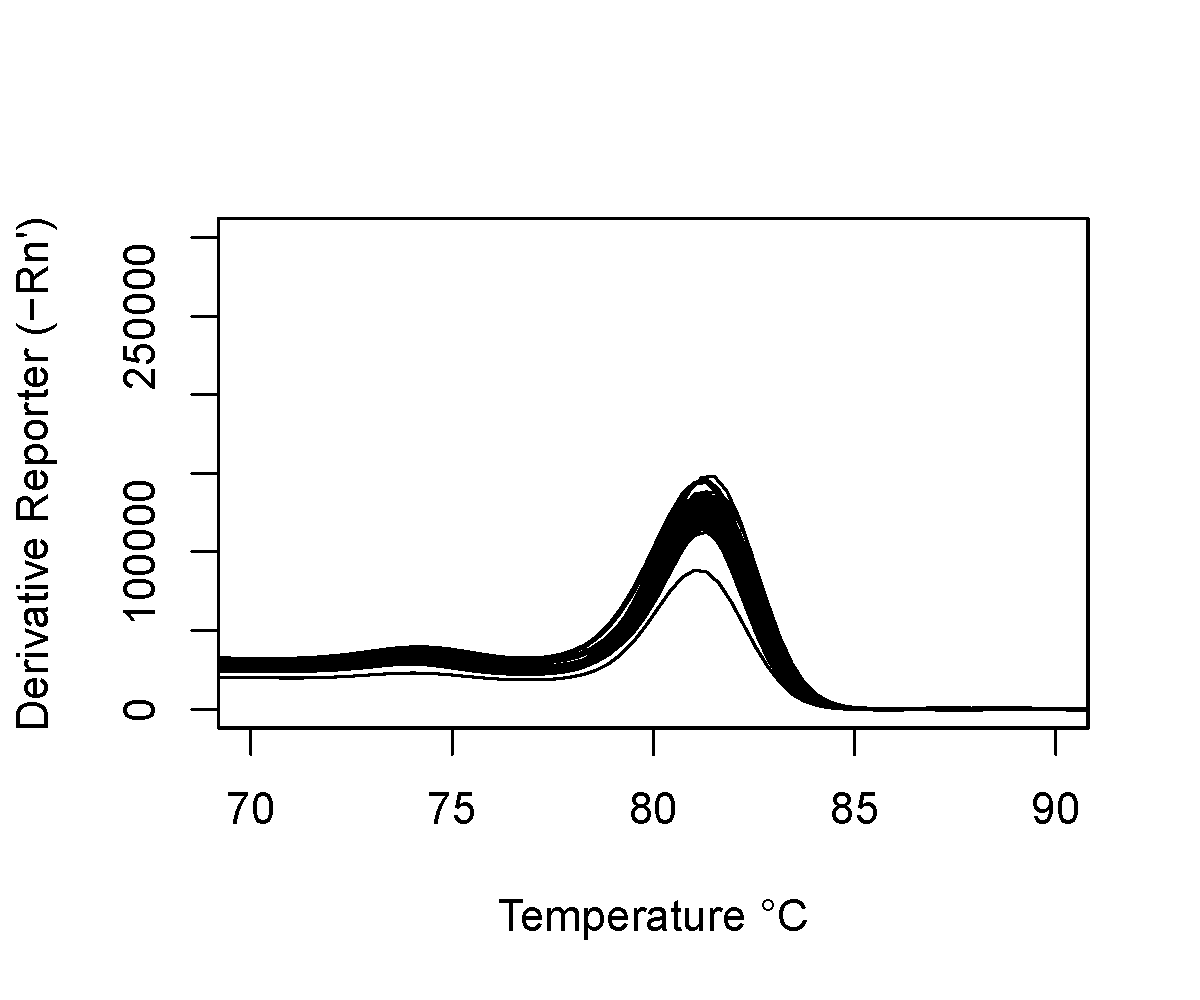*** | ***PSB33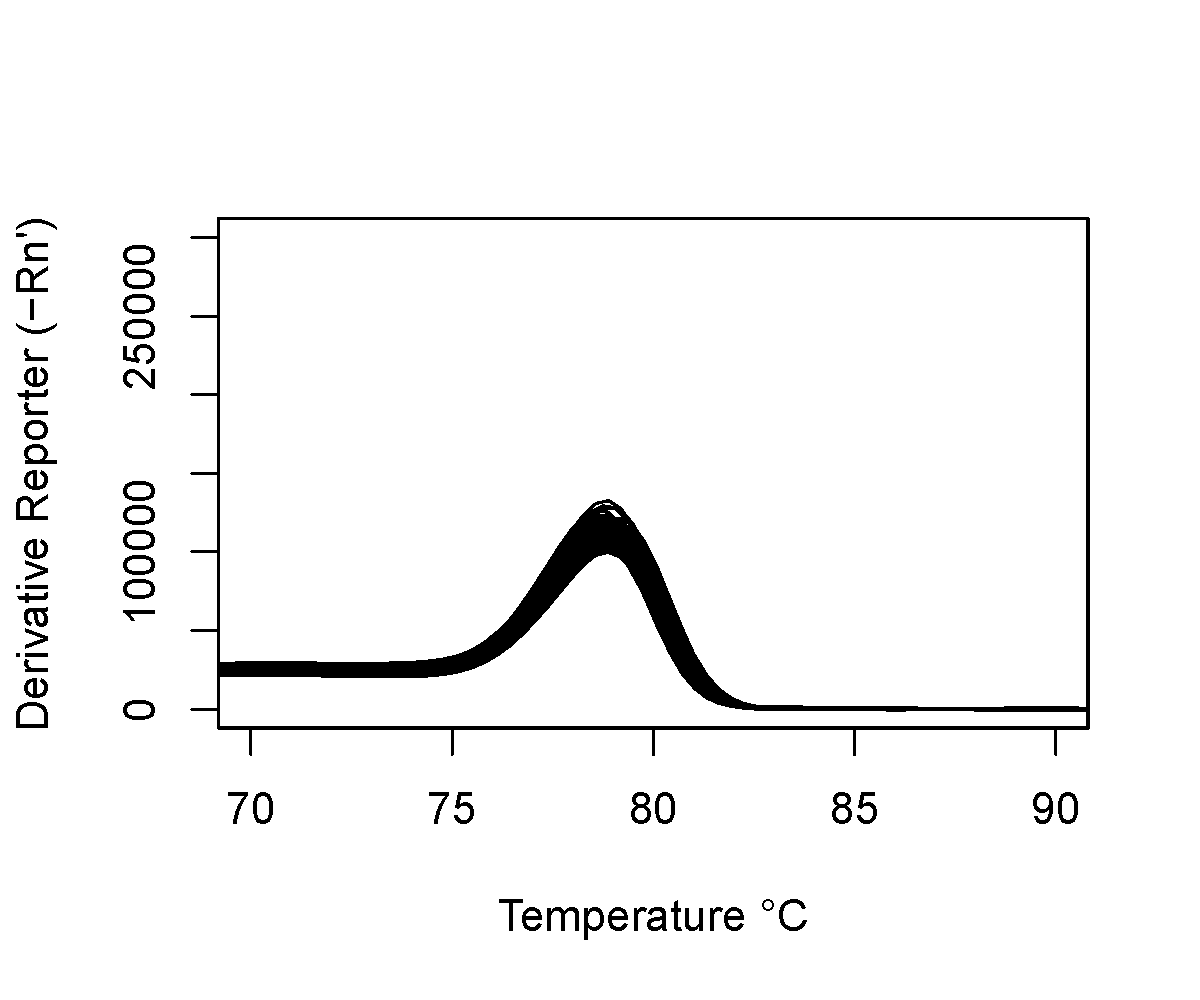*** |
|  | ***HISTONE H3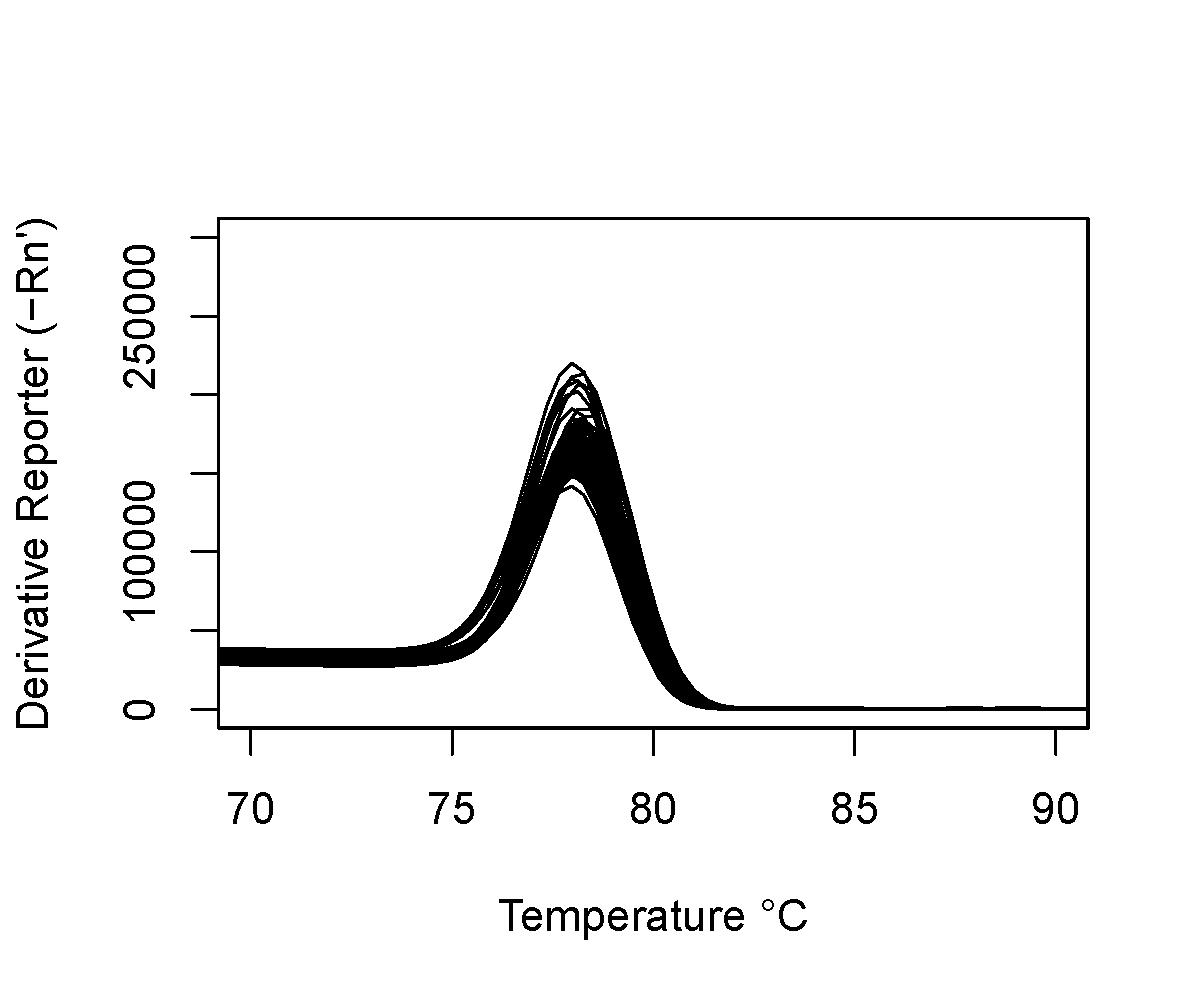*** | ***NdhO 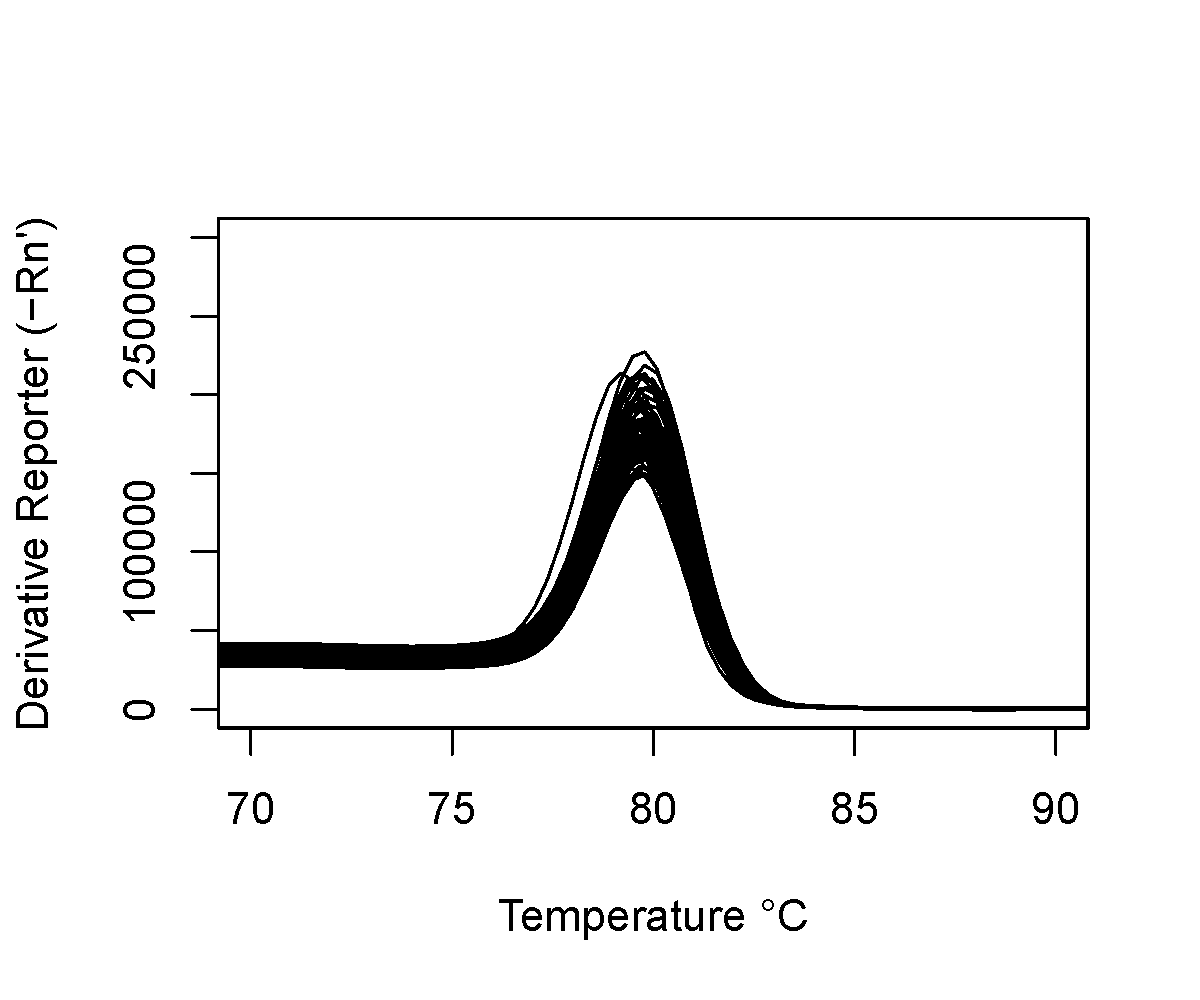*** |
|  | ***TUA5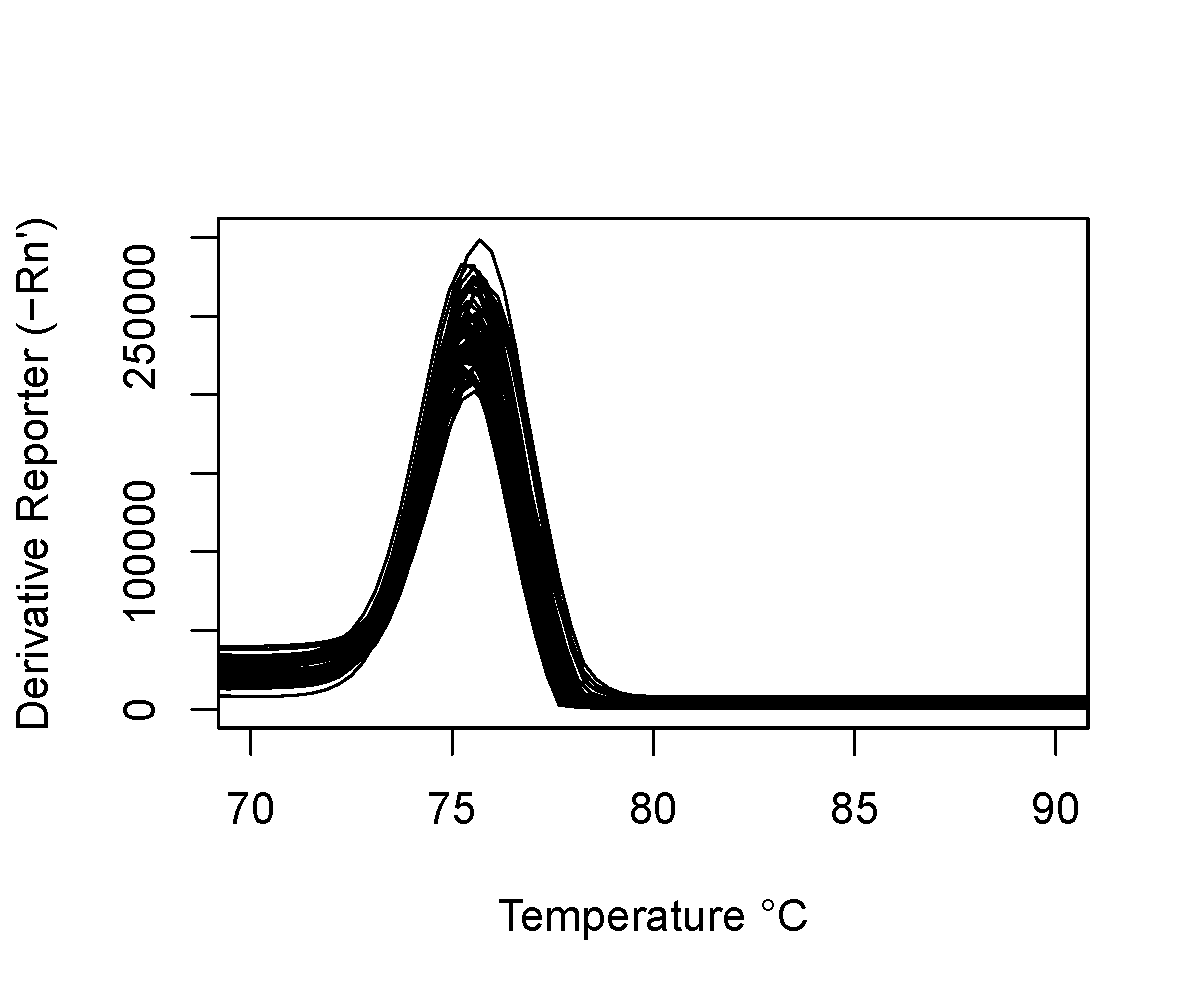*** | ***18srRNA 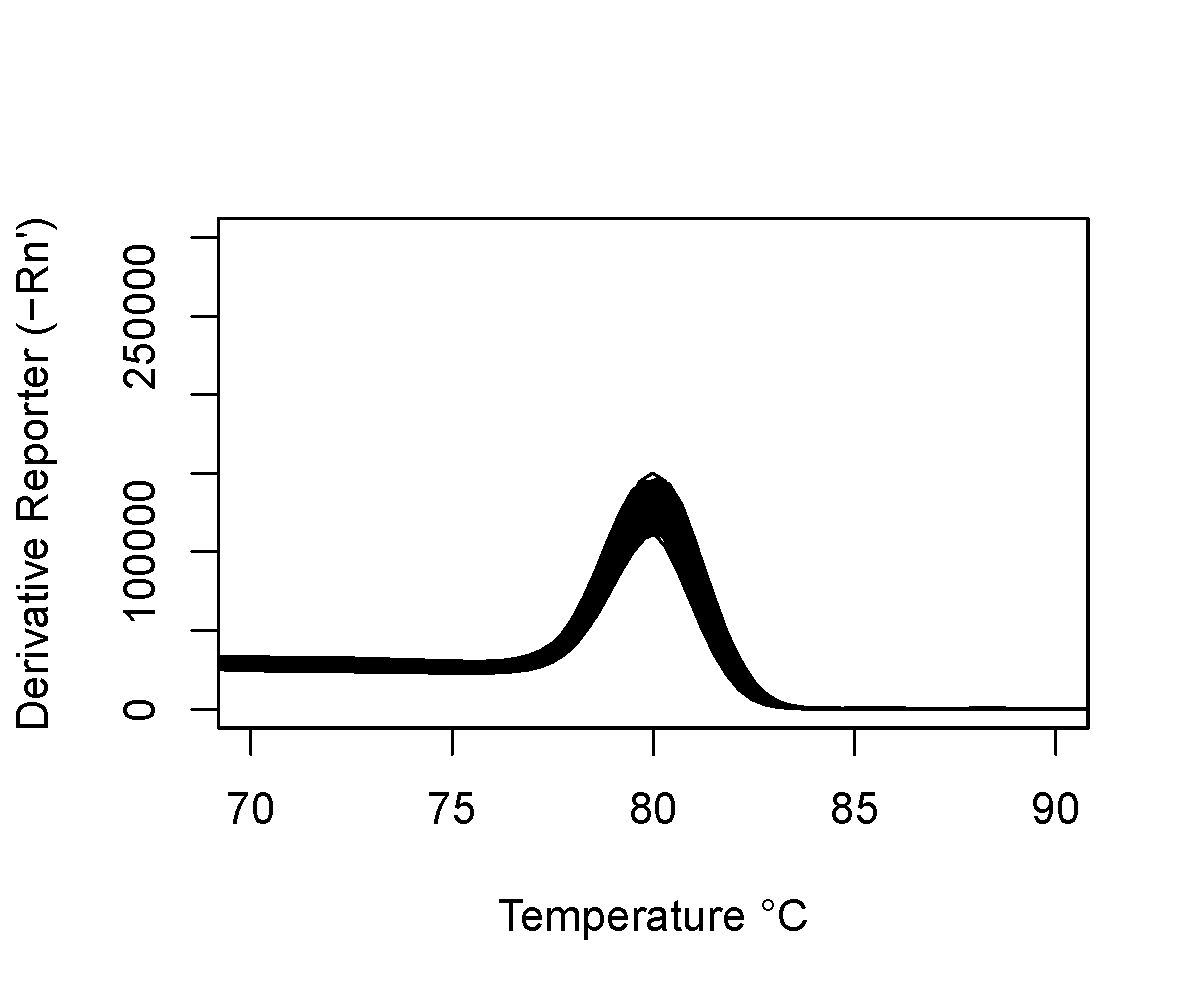*** |
|  | ***CAC 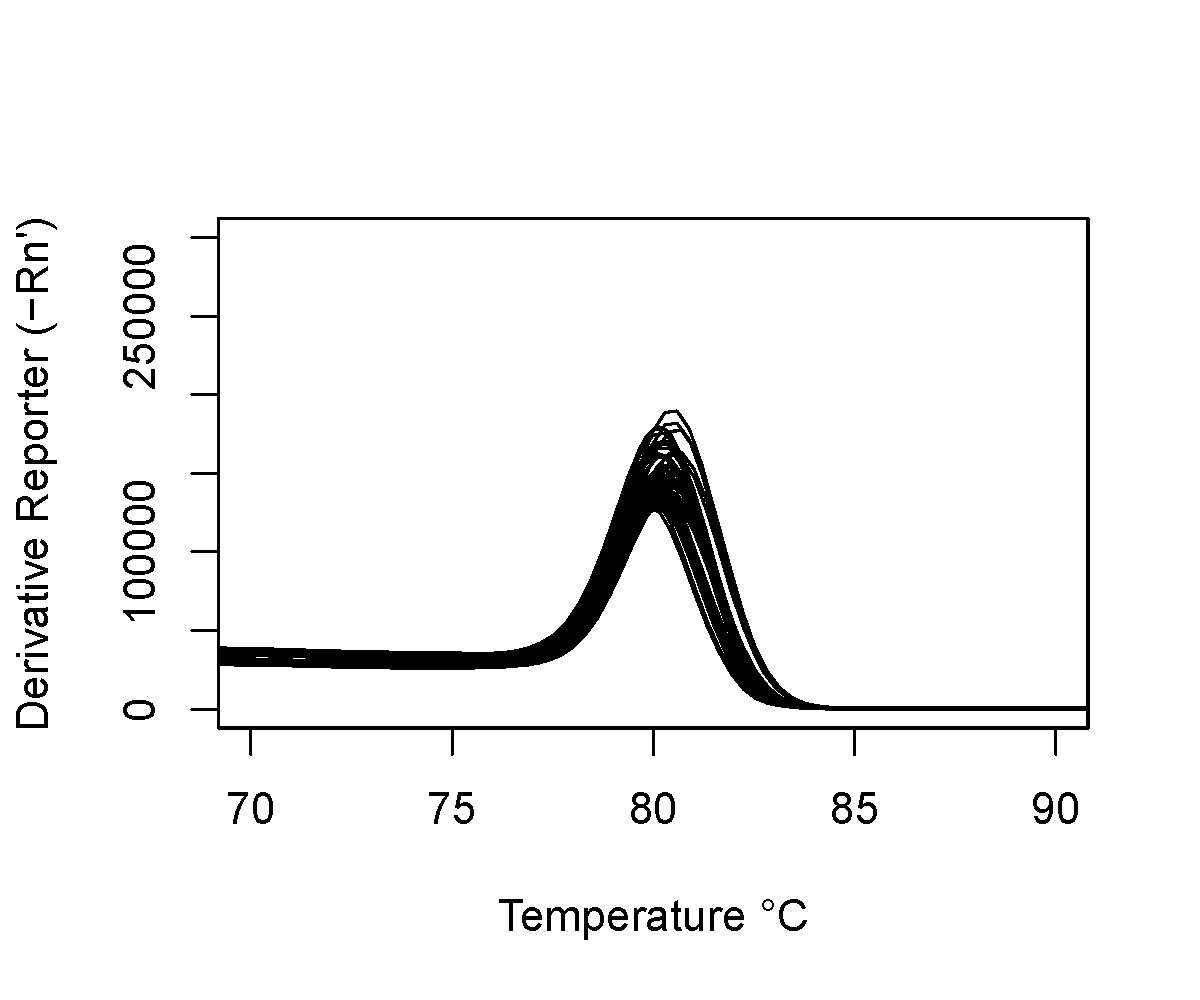*** | ***SAND 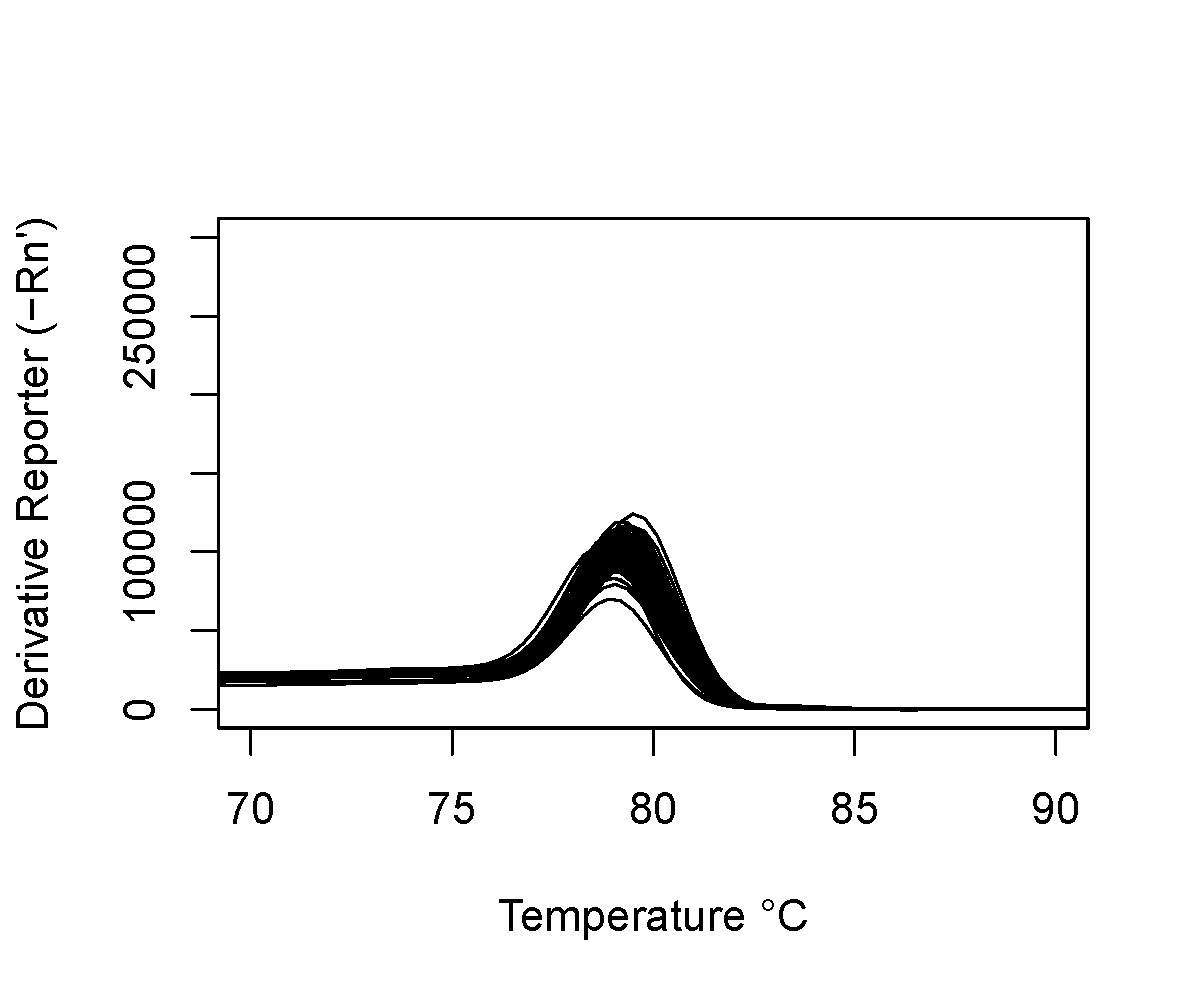*** |
|  | ***HSP81.2/90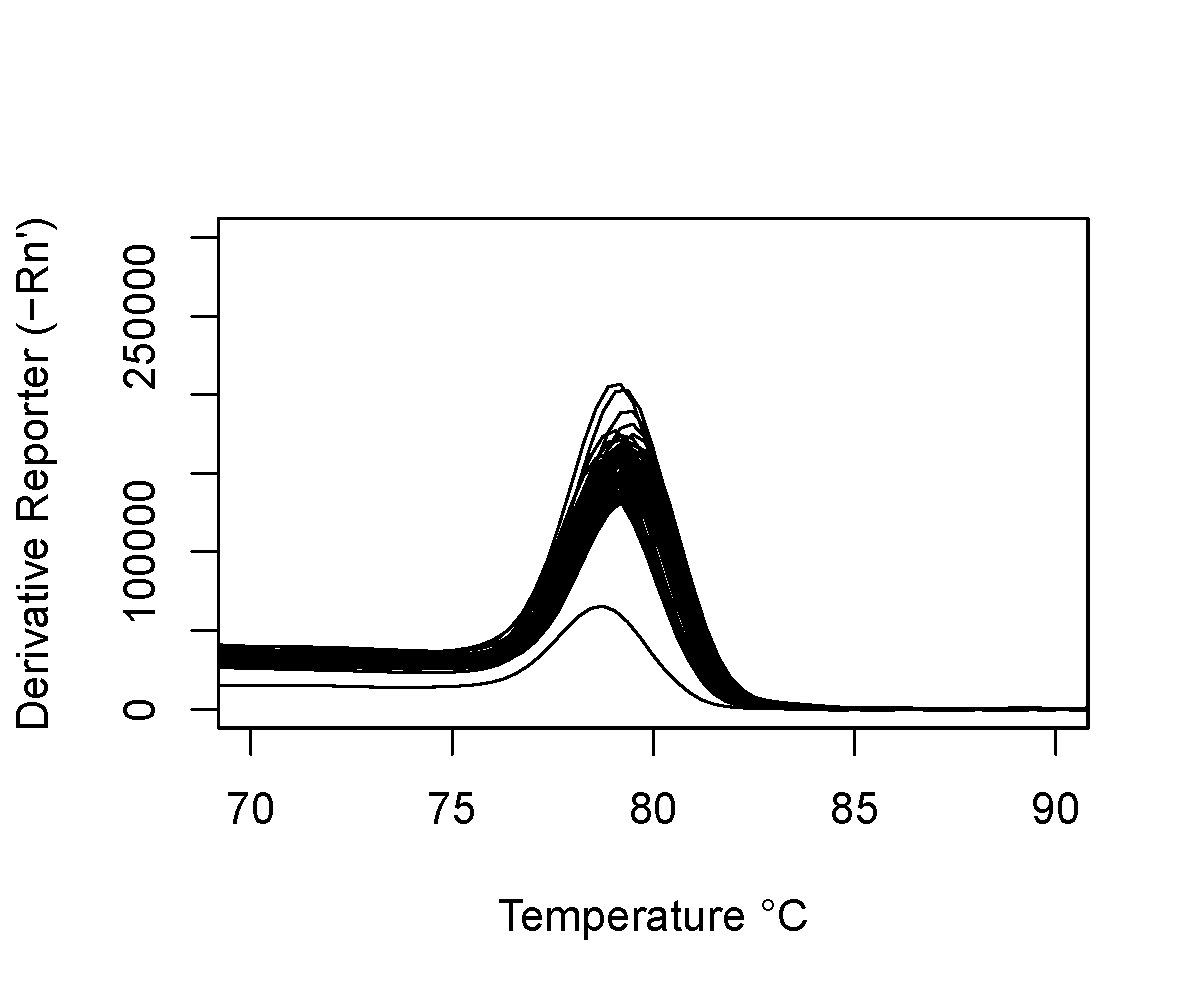*** | ***RD29A***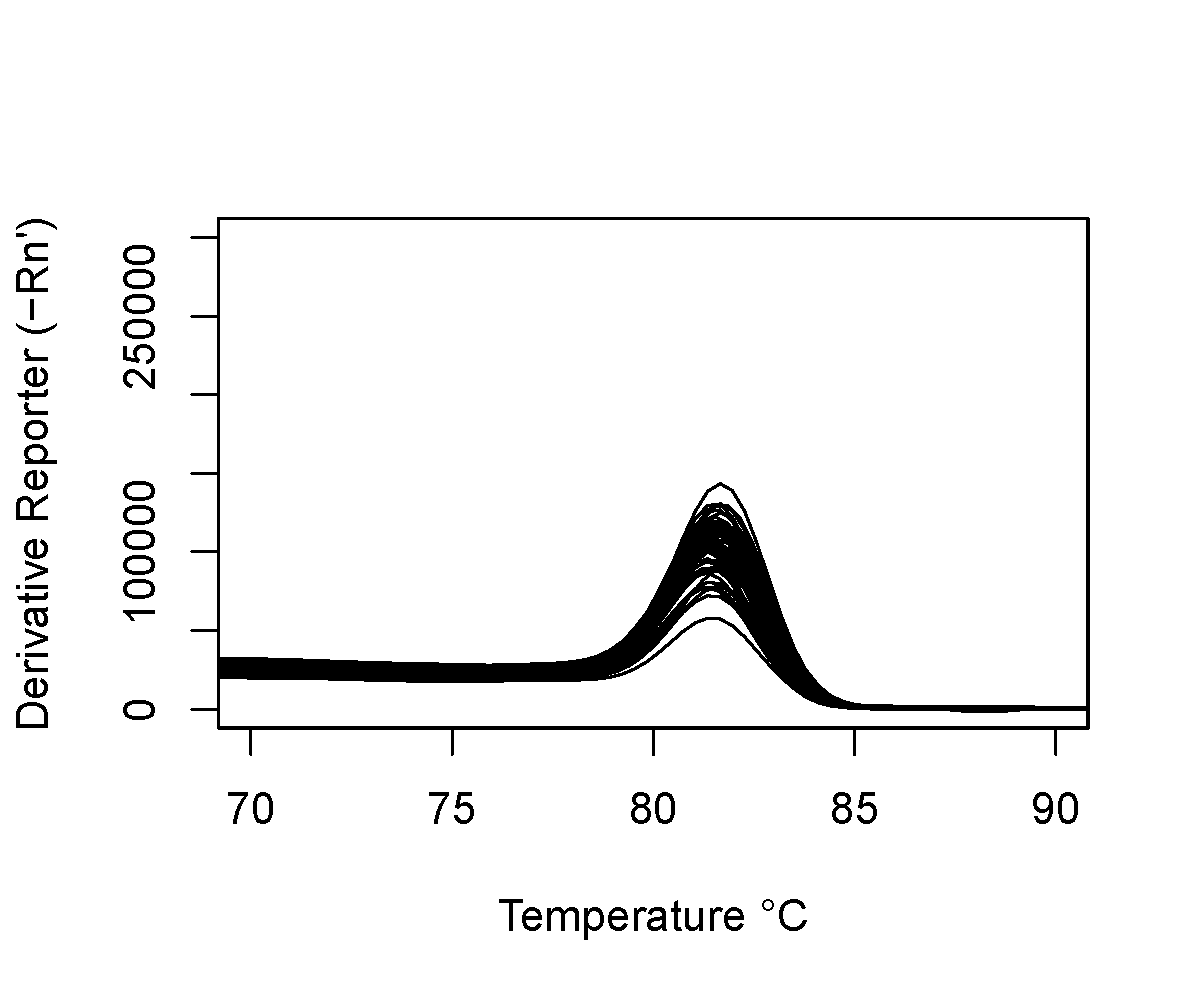 |
|  | ***TSPO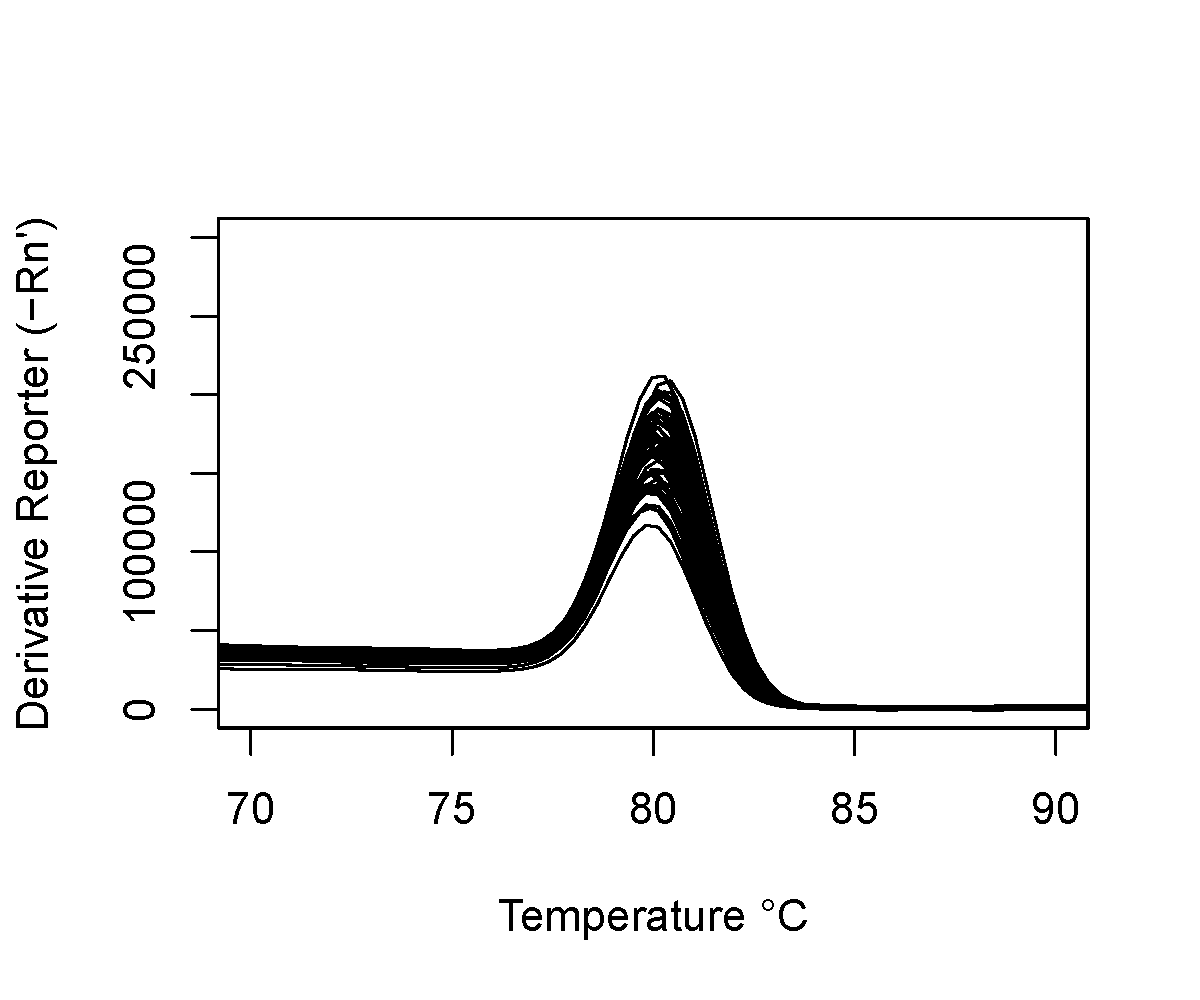*** | ***GAox1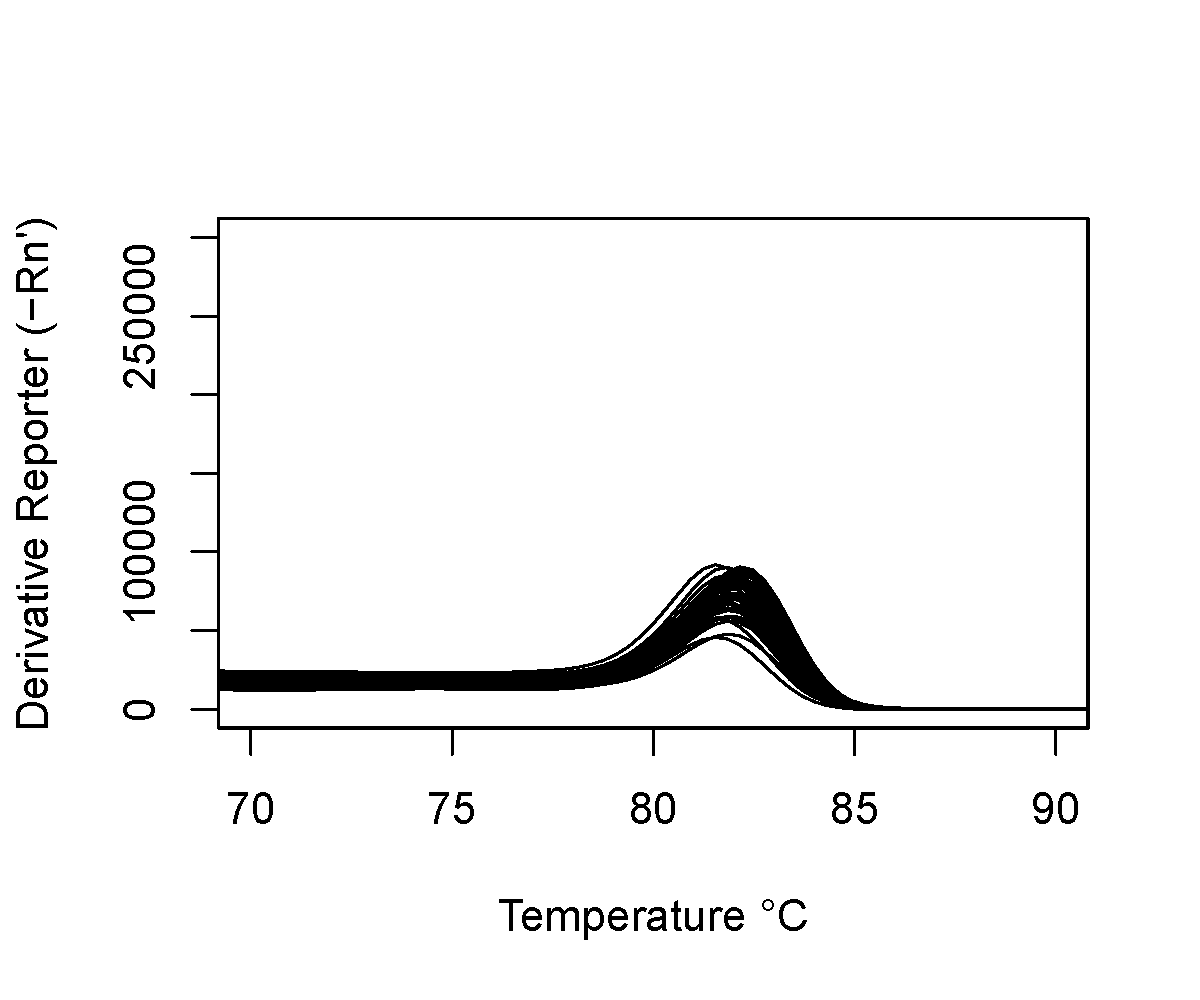*** |

**S1 Fig. Amplicon analysis of candidate reference genes and stress/hormone responsive genes.**

A - gel electrophoresis picture of a 2% agarose gel, marker: GeneRuler 100 bp Plus (Thermo Fisher Scientific). B – primer melting curves.
