# Supplementary material for: Selection and validation of reference genes for quantitative Real-Time PCR in Arabis alpina
Source: PLoS One. 2019 Mar 4;14(3):e0211172. doi: 10.1371/journal.pone.0211172 (PMC6398851; doi:10.1371/journal.pone.0211172)
Supplement: S2 Fig — (DOCX) [file pone.0211172.s003.docx]

| ***ATPase*** | ***THIOREDOXIN*** |
| --- | --- |
| ***HCF*** | ***EIF4a*** |
| ***RAN3*** | ***UBQ10*** |
| ***ACTIN 2*** | ***PSB33*** |
| ***HISTONE H3*** | ***NdhO*** |
| ***TUA5*** | ***18srRNA*** |
| ***CAC*** | ***SAND*** |
| ***HSP81.2/90*** | ***RD29A*** |
| ***TSPO*** | ***GAox1*** |

**S2 Fig. Standard curves of candidate reference genes and stress/hormone responsive genes.**
