# Supplementary material for: Selection and validation of reference genes for quantitative Real-Time PCR in Arabis alpina
Source: PLoS One. 2019 Mar 4;14(3):e0211172. doi: 10.1371/journal.pone.0211172 (PMC6398851; doi:10.1371/journal.pone.0211172)
Supplement: S2 Table — (DOCX) [file pone.0211172.s004.docx]

**S2 Table. Ranking of gene expression stability under abiotic stress conditions and the combination of all treatments.** Genes were ranked using the three commonly used statistical algorithms NormFinder, BestKeeper and geNorm. The stability value describes the variance (NormFinder and geNorm) or standard deviation (BestKeeper).

|  | Rank |  | NormFinder | |  | BestKeeper | |  | geNorm | |
| --- | --- | --- | --- | --- | --- | --- | --- | --- | --- | --- |
|  |  |  | Gene | Stability |  | Gene | Stability |  | Gene | Stability |
| abiotic stress | 1 |  | *THIOREDOXIN* | 0.09 |  | *ACTIN 2* | 1.44 |  | *THIOREDOXIN* | 0.54 |
|  | 2 |  | *PSB33* | 0.16 |  | *UBQ10* | 1.48 |  | *CAC* | 0.58 |
|  | 3 |  | *HCF* | 0.19 |  | *18srRNA* | 1.58 |  | *TUA5* | 0.59 |
|  | 4 |  | *NdhO* | 0.22 |  | *CAC* | 1.60 |  | *PSB33* | 0.60 |
|  | 5 |  | *CAC* | 0.23 |  | *TUA5* | 1.62 |  | *HCF* | 0.61 |
|  | 6 |  | *TUA5* | 0.23 |  | *THIOREDOXIN* | 1.69 |  | *ATPase* | 0.62 |
|  | 7 |  | *ATPase* | 0.24 |  | *HCF* | 1.70 |  | *NdhO* | 0.63 |
|  | 8 |  | *HISTONE H3* | 0.30 |  | *SAND* | 1.71 |  | *ACTIN 2* | 0.65 |
|  | 9 |  | *ACTIN 2* | 0.30 |  | *ATPase* | 1.77 |  | *HISTONE H3* | 0.69 |
|  | 10 |  | *18srRNA* | 0.32 |  | *RAN3* | 1.77 |  | *18srRNA* | 0.70 |
|  | 11 |  | *UBQ10* | 0.36 |  | *PSB33* | 1.88 |  | *SAND* | 0.71 |
|  | 12 |  | *SAND* | 0.39 |  | *NdhO* | 1.90 |  | *UBQ10* | 0.76 |
|  | 13 |  | *EIF4a* | 0.41 |  | *HISTONE H3* | 1.90 |  | *EIF4a* | 0.79 |
|  | 14 |  | *RAN3* | 0.49 |  | *EIF4a* | 1.94 |  | *RAN3* | 0.87 |
|  | 15 |  | *HSP81.2/90* | 1.18 |  | *HSP81.2/90* | 3.23 |  | *HSP81.2/90* | 1.75 |
| all treatments | 1 |  | *CAC* | 0.20 |  | *UBQ10* | 1.45 |  | *CAC* | 0.63 |
|  | 2 |  | *TUA5* | 0.24 |  | *18srRNA* | 1.54 |  | *TUA5* | 0.65 |
|  | 3 |  | *ACTIN 2* | 0.26 |  | *ACTIN 2* | 1.57 |  | *ACTIN 2* | 0.69 |
|  | 4 |  | *ATPase* | 0.27 |  | *RAN3* | 1.59 |  | *ATPase* | 0.69 |
|  | 5 |  | *PSB33* | 0.30 |  | *CAC* | 1.70 |  | *THIOREDOXIN* | 0.70 |
|  | 6 |  | *THIOREDOXIN* | 0.32 |  | *SAND* | 1.72 |  | *PSB33* | 0.71 |
|  | 7 |  | *HISTONE H3* | 0.32 |  | *TUA5* | 1.76 |  | *SAND* | 0.74 |
|  | 8 |  | *HCF* | 0.34 |  | *HISTONE H3* | 1.76 |  | *HCF* | 0.74 |
|  | 9 |  | *SAND* | 0.34 |  | *EIF4a* | 1.81 |  | *HISTONE H3* | 0.78 |
|  | 10 |  | *EIF4a* | 0.36 |  | *HCF* | 1.87 |  | *EIF4a* | 0.79 |
|  | 11 |  | *UBQ10* | 0.44 |  | *ATPase* | 1.87 |  | *NdhO* | 0.83 |
|  | 12 |  | *NdhO* | 0.45 |  | *THIOREDOXIN* | 1.94 |  | *UBQ10* | 0.89 |
|  | 13 |  | *18srRNA* | 0.48 |  | *PSB33* | 1.97 |  | *18srRNA* | 0.91 |
|  | 14 |  | *RAN3* | 0.52 |  | *NdhO* | 2.13 |  | *RAN3* | 0.96 |
|  | 15 |  | *HSP81.2/90* | 1.13 |  | *HSP81.2/90* | 2.66 |  | *HSP81.2/90* | 1.70 |
